# Repurposing Product Nkabinde for Hepatitis B Virus Therapy: A Network Pharmacology and Molecular Docking Investigation

**DOI:** 10.3390/ph19040627

**Published:** 2026-04-16

**Authors:** Samuel Chima Ugbaja, Siphathimandla Authority Nkabinde, Magugu Nkabinde, Nceba Gqaleni

**Affiliations:** 1 Traditional Medicine, School of Medicine, University of KwaZulu Natal, Durban 4000, South Africa; nceba5850@gmail.com (S.A.N.); magugun@webmail.com (M.N.); 2 African Health Research Institute (AHRI), 719 Umbilo Road, Durban 4001, South Africa

**Keywords:** Product Nkabinde, Hepatitis B virus, drug repurposing, polyherbal formulation, network pharmacology, molecular docking

## Abstract

**Background**: Hepatitis B virus (HBV) infection continues to be a major public health concern, especially in sub-Saharan Africa, where widespread epidemics and restricted availability of long-term antiviral therapies result in higher mortality and morbidity rates. Drug repurposing represents a strategic approach to accelerate the discovery of effective therapies by leveraging agents with demonstrated antiviral and immunomodulatory activity. Product Nkabinde (PN) is a patented African polyherbal formulation initially developed for the treatment of HIV. Recent experimental studies demonstrate PN’s potent anti-HIV activity and significant immunomodulatory effects in human immune cells, implicating host-directed mechanisms relevant to chronic viral infections. This study combines an integrative application of network pharmacology and molecular docking to evaluate the repurposing potential of PN as a multi-target agent in HBV. **Method**: Bioactive components of PN were screened, and compound-associated targets were intersected with HBV-associated genes (proteins) to construct a protein–protein interaction (PPI) network. Topological analysis identified 10 hub targets (STAT1, STAT3, SRC, HCK, EGFR, SYK, PIK3CA, PIK3CB, PIK3R1, and PTPN11). Gene Ontology and KEGG pathway enrichment were performed with an FDR cut-off < 0.05. Significantly enriched pathways included JAK–STAT signaling, chemokine signaling, EGFR-TKI resistance, PI3K complex signaling, and viral infection pathways, particularly those related to Kaposi sarcoma virus and HSV-1, indicating immunoregulatory and antiviral roles. Molecular docking was performed using AutoDock Vina 1.1.2 to evaluate binding affinity and interaction mode of key PN phytochemicals against the hub proteins, and results were compared to their respective co-crystallized ligands. **Results**: Molecular docking indicated that major phytochemicals from PN exhibited significant binding affinities across all 10 hub host targets, typically outperforming or closely matching their respective co-crystallized ligands. The strongest contacts were observed for β-sitosterol–PIK3CB (−14.2 kcal/mol) and oleanolic acid–SYK (−14.0 kcal/mol), which were significantly stronger than the co-crystallized ligands (−7.9 and −8.3 kcal/mol, respectively), indicating robust stabilization within catalytic and regulatory pockets. Procyanidin B2 toward HCK (−10.5 vs. −7.9 kcal/mol) and PIK3CA (−9.5 vs. −7.3 kcal/mol), quercetin toward PIK3R1 (−10.6 vs. −8.2 kcal/mol) and PTPN11 (−9.2 vs. −7.5 kcal/mol), rutin toward SRC (−10.5 vs. 7.8 kcal/mol), and diosgenin toward EGFR (−9.4 vs. 8.4 kcal/mol). Procyanidin B2 maintained robust multi-hydrogen bonding networks, demonstrating significant binding, despite STAT1 and STAT3 docking showing identical affinities to co-crystals. Conserved hydrogen bonds, π–cation interactions, and significant hydrophobic packing at ATP-binding clefts and regulatory domains supported these interaction patterns, indicating competitive suppression of host signaling nodes taken over by HBV. **Conclusions**: Together, these results demonstrate that the components of PN possess strong multitarget binding capabilities across the PI3K/AKT, JAK–STAT, SRC-family kinase, EGFR, and SYK pathways, supporting their potential repurposing as host-directed HBV therapeutics with the ability to impede immune evasion, viral persistence, and HBV-associated oncogenic progression.

## 1. Introduction

Hepatitis B virus (HBV) infection remains a major public health concern, with over 254 million individuals living with chronic infection and over 1.1 million annual deaths, mostly owing to cirrhosis and hepatocellular carcinoma (HCC) [1]. Sub-Saharan Africa and the Western Pacific, where perinatal and early childhood transmission predominate, are disproportionately affected by chronic HBV [2]. Due to the persistence of covalently closed circular DNA (cccDNA) and immune dysfunction, nucleos(t)ide analogues (NAs) like tenofovir and entecavir rarely achieve functional cure, which is defined as sustained hepatitis B surface antigen (HBsAg) loss and undetectable viral DNA off therapy. Patients are still at risk of fibrosis development and HCC, even with extended therapy [3].

Drug repurposing accelerates the development of new treatments by leveraging substances with proven safety and established pharmacological properties [3]. Furthermore, due to their similar dependence on host replication pathways, a number of antiretroviral medications that were first created for HIV, such as lamivudine and tenofovir, were successfully adapted for HBV [4,5]. To reduce resistance and improve immune-mediated control, modern repurposing efforts are increasingly focusing on host-targeted, multi-mechanistic drugs that simultaneously alter immune signaling and viral–host interactions [2,6]. A wide range of structurally diverse molecules with multitarget potential, including antiviral, anti-inflammatory, antioxidant, and immunomodulatory properties, can be found in natural products and traditional medicines. Flavonoids, terpenoids, and polyphenols are examples of phytochemicals that have demonstrated efficacy against various viruses, often by modulating host cell signaling and immunological pathways rather than directly inhibiting viral enzymes [7].

Product Nkabinde (PN) is a proprietary African polyherbal mixture that has been historically used for immune support and antiviral therapy. A study by Mngomezulu et al. [8] demonstrated potent in vitro anti-HIV activity, with PN inhibiting HIV-1 replication in a HeLa-derived reporter cell line (TZM-bl) and in human peripheral blood mononuclear cells (PBMCs), achieving up to 96% viral suppression and showing synergistic effects with established antiretrovirals [8]. Another study by Setlhare et al. [9] confirmed PN’s immunomodulatory effects, including induction of TNF-α, IL-1α, IL-10, and MIP-1β, as well as CD4^+^ T cell activation [9]. PN’s immunomodulatory profile is consistent with the host dysregulation observed in chronic HBV infection, in which persistence and disease progression are attributed to impaired innate responses, natural killer (NK) cell dysfunction, T cell exhaustion, and altered cytokine signaling [10]. Instead of merely extrapolating efficacy from one virus to another, this molecular commonality between HIV-related host pathways and those disrupted in HBV offers a compelling biological justification for repurposing PN as a host-directed adjuvant in HBV therapy.

Network pharmacology, which combines systems biology, pharmacology, and bioinformatics, is particularly useful for analyzing the multi-target mechanisms of complex formulations, such as PN. This approach enables the identification of common nodes between disease gene networks and phytochemical targets, as well as the ranking of key hub proteins in host signaling pathways involved in HBV pathogenesis. Instead of using virus-specific enzymatic methods, this method offers a systems-level framework for logical repurposing based on shared host dependencies [11]. The selection of twenty-seven (27) phytochemicals that are associated with Product Nkabinde (PN) was derived from previous experimental chemical profiling and compound isolation studies conducted by our collaborators at the University of Pretoria. Tembeni et al. [12] implemented HPLC-based purification, isolation, and structural characterization methodologies to identify bioactive constituents from the medicinal plant components of PN, specifically Gnidia sericocephala, Senna italica, and Sclerocarya species, which serve as the fundamental botanical foundation of the formulation. The authors reported the isolation and characterization of numerous diterpenes, flavonoids, triterpenoids, and polyphenolic compounds, all of which have been shown to possess antiviral and immunomodulatory properties [12]. Maharaj et al. [13], on the other hand, further characterized and validated anti-HIV and latency-reversing agents during the formulation of the PN herbal mixture. The 27 compounds examined in this study are experimentally identified and structurally validated phytochemicals that have been consistently documented in profiling efforts. They were selected based on confirmed presence in PN constituent plants, reproducibility in independent isolation studies, and biological significance to host immune and antiviral signaling pathways. Due to the intrinsic compositional heterogeneity of polyherbal formulations, a presence-based component selection strategy was implemented instead of abundance-weighted prioritization, consistent with recognized network pharmacology methodologies for complex herbal medications. This method is suitable for examining multitarget, host-directed mechanisms of action that constitute the therapeutic rationale of PN [13]. Another recent study by our research group, “Molecular Investigation of Product Nkabinde in HIV Therapy: A Network Pharmacology and Molecular Docking Approach,” serves as the basis for this repurposing study [14]. In this study, we employ an integrative network pharmacology and molecular docking platform to investigate how PN’s constituent phytochemicals interact with host targets implicated in HBV biology. This study aims to identify multitarget mechanisms through which PN may influence immune regulation, inflammatory signaling, and viral persistence in chronic HBV infection by intersecting PN targets with HBV-associated genes, constructing protein–protein interaction networks, and evaluating key host hub proteins. Furthermore, this study provides a mechanistic and evidence-based argument for repurposing PN as a supplementary host-directed treatment strategy in HBV management.

## 2. Results and Discussion

### 2.1. Intersection Analysis of HBV and PN Target Proteins

**Figure 1:**
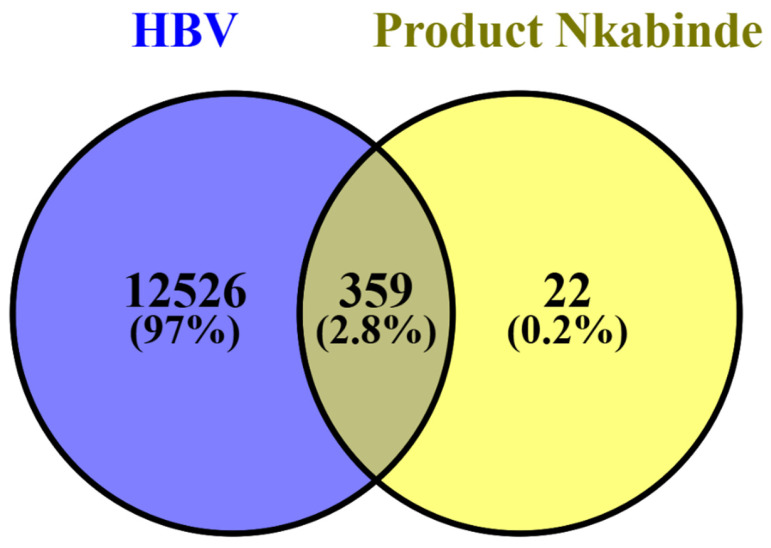
A VENN illustration of the degree of overlap between proteins associated with HBV pathogenesis and those predicted for PN.

The Venn diagram in Figure 1 illustrates the degree of overlap between proteins associated with HBV pathogenesis and those predicted for PN, suggesting the potential for repurposing the product. A total of 12,526 targets are specifically related to HBV, whereas 22 targets are exclusive to PN, demonstrating that the formulation has a targeted yet pharmacologically relevant profile [15]. Most importantly, given the current knowledge that multi-target modulation is beneficial in HBV therapy due to viral persistence and host immune dysregulation, the intersection of 359 shared targets (2.8%) represents a significant convergence zone where PN may modulate biological processes directly linked to HBV infection and host–virus interactions [15]. Furthermore, network pharmacology, a multidisciplinary strategy that combines computational methods with systems biology to discover drug–target–disease interactions, has recently been emphasized as a potent technique for identifying multi-target mechanisms and aiding with drug repurposing, including for complex diseases and polypharmacological natural products [11].

The shared target set is strategically important as the mechanistic focus for subsequent pathway enrichment and molecular docking analyses. The convergence of targets highlighted by this diagram is consistent with emerging evidence that natural products can exert antiviral effects by engaging multiple host and viral pathways, as reported in recent research into natural antivirals against chronic HBV [16].

### 2.2. Interaction Network of Shared HBV–PN Targets

The interaction network shown in Supplementary Figure S11 reveals a dense interactome of the 359 shared targets between HBV-related pathways and PN-predicted targets, highlighting that these targets are not isolated elements but components of an integrated biological system involving host cellular functions [17]. The high connectivity of this network points to core hubs and modules that likely coordinate important immune regulatory processes and signaling pathways associated with HBV chronicity. This finding aligns with a recent network pharmacology study that identifies key interconnected proteins as drivers of HBV-associated disease mechanisms and therapeutic responses [18].

Using such interaction network maps enhances the identification of critical nodes that are both pharmacologically tractable and disease-relevant for drug discovery. This is consistent with the network medicine paradigm, which holds that perturbations of central network elements can improve repurposing success rates and shift dysregulated systems toward homeostasis [19]. As a result, the network’s extensive interconnectivity facilitates the prioritization of hubs for molecular docking and functional enrichment, laying the groundwork for a mechanistic explanation of how the phytochemical components of PN may interact with and alter important protein complexes in the context of HBV disease.

### 2.3. Identifying HBV-PN Hub Proteins

**Figure 2:**
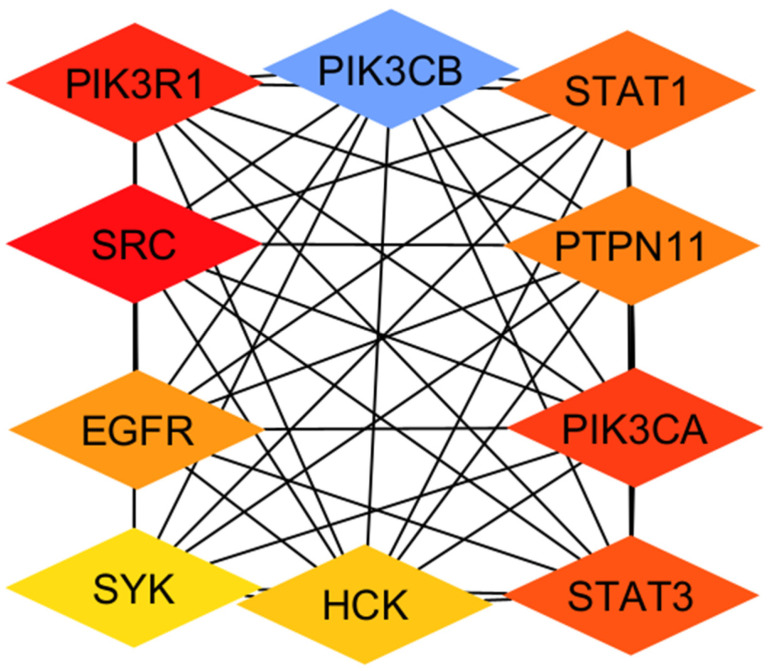
Diagram of the identified HBV-PN Hub Proteins.

Figure 2 below illustrates the key hub proteins, including PIK3R1, PIK3CB, STAT1, PTPN11, PIK3CA, STAT3, HCK, SYK, EGFR, and SRC, retrieved from the common HBV-PN protein–protein interaction network. These hubs have the potential to be strategic therapeutic leverage points because they reflect biological processes known to mediate the hepatocellular response to viral infection, immune signaling, and inflammation. The inclusion of STAT1 and STAT3, key transcriptional regulators of interferon and cytokine signaling, is consistent with recent findings that modulation of the JAK–STAT axis affects HBV persistence and antiviral immunity, making these genes desirable targets for multi-target intervention [20].

Collectively, these hubs connect on pathways involving growth factor signaling, immune regulation, and cell survival/apoptosis, providing mechanistic insight into how PN’s multi-component action could interact with key host networks disrupted in HBV infection. This network framework highlights a multi-target repurposing strategy whereby modulating central nodes can shift dysregulated host–virus interplay toward therapeutic benefit. Host-directed therapeutic strategies have emerged as promising complementary approaches in chronic viral infections, specifically where conventional antiviral therapies fail to eradicate viral reservoirs or fully restore immune function. While nucleos(t)ide analogue therapies effectively suppress HBV replication, they do not eliminate covalently closed circular DNA (cccDNA) and rarely achieve functional cure due to persistent viral antigenemia and immune dysregulation. This has motivated exploration of therapies that modulate host dependency factors and immune signaling pathways critical to HBV persistence and pathogenesis. Recent reviews underscore the complexity of HBV–host interactions and propose host factors as viable therapeutic targets to augment the efficacy of direct-acting antivirals and overcome the limitations of current regimens [21]. Moreover, host metabolic and signaling pathways exploited by the virus may represent additional avenues for intervention, supporting host-directed multi-target strategies, as explored in this study [22]. Collectively, these perspectives align with the integrative repurposing framework used here, positioning PN as a candidate for modulating host pathways to potentially enhance HBV control alongside existing therapies.

### 2.4. Functional Enrichment

**Figure 3:**
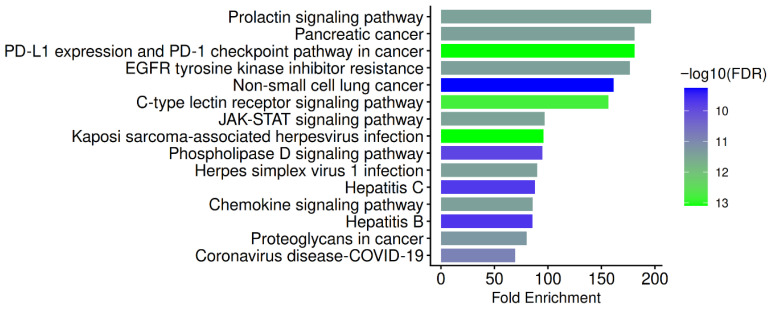
KEGG pathway enrichment analysis of PN–HBV hub genes. Bar plot showing the top significantly enriched KEGG pathways associated with the ten identified PN–HBV hub genes (STAT1, STAT3, SRC, HCK, EGFR, SYK, PIK3CA, PIK3CB, PIK3R1, and PTPN11). Pathways include prolactin signaling, pancreatic cancer, PD-L1 expression and PD-1 checkpoint pathway, EGFR-tyrosine kinase inhibitor resistance, non-small cell lung cancer, C-type lectin receptor signaling, JAK–STAT signaling, Kaposi sarcoma-associated herpesvirus infection, phospholipase D signaling, herpes simplex virus 1 infection, hepatitis C, chemokine signaling, hepatitis B, proteoglycans in cancer, and COVID-19. Bar length represents fold enrichment, while color indicates statistical significance expressed as −log10(FDR). The results demonstrate that PN targets host immune, inflammatory, oncogenic, and viral infection pathways relevant to HBV persistence and pathogenesis.

**Figure 4:**
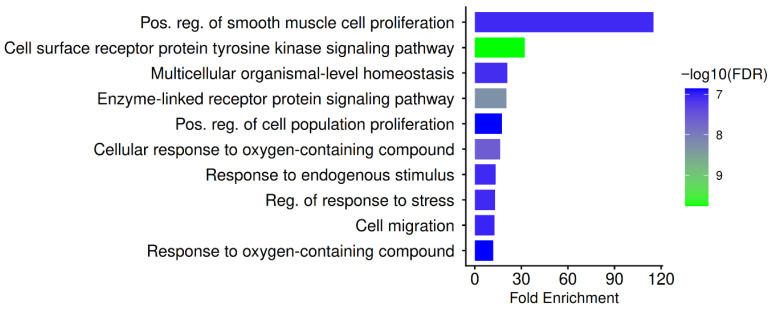
GO Biological Process enrichment of PN-HBV hub gene. The figure depicts the major enriched biological processes associated with PN-HBV hub genes. Significantly represented terms include positive regulation of smooth muscle cell proliferation, cell surface receptor protein tyrosine kinase signaling pathway, multicellular organismal-level homeostasis, enzyme-linked receptor protein signaling pathway, positive regulation of cell population proliferation, cellular response to oxygen-containing compound, response to endogenous stimulus, regulation of response to stress, cell migration, and response to oxygen-containing compound. Fold enrichment is indicated by bar length, and −log10(FDR) is represented by the color scale. Enriched processes reflect cellular proliferation, stress responses, and receptor-mediated immune signaling implicated in HBV-related inflammation, fibrosis, and hepatocarcinogenesis.

**Figure 5:**
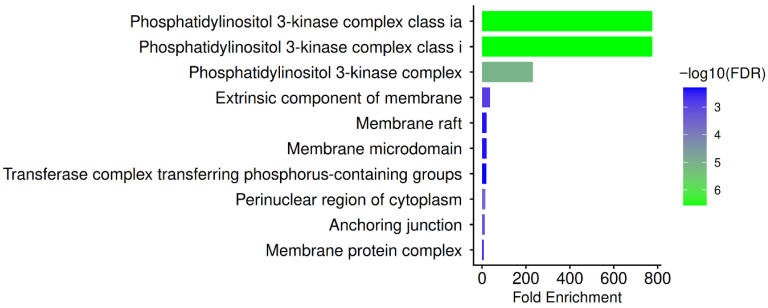
GO Cellular Component enrichment of PN-HBV hub genes. Bar plot showing enriched cellular components associated with the PN-HBV hub genes. Highly enriched terms include phosphatidylinositol 3-kinase complex class IA, phosphatidylinositol 3-kinase complex class I, phosphatidylinositol 3-kinase complex, extrinsic component of membrane, membrane raft, membrane microdomain, transferase complex transferring phosphorus-containing groups, perinuclear region of cytoplasm, anchoring junction, and membrane protein complex. The predominance of PI3K complexes and membrane-associated domains indicates localization of hub targets in lipid-raft signaling platforms involved in viral entry, cytokine signaling, and hepatocyte survival pathways exploited during HBV infection.

**Figure 6:**
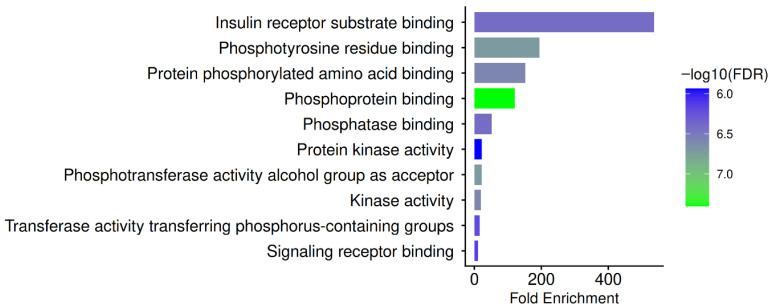
GO Molecular Function enrichment of PN-HBV hub genes. This figure displays the significantly enriched molecular functions of the PN-HBV hub genes. Enriched terms include insulin receptor substrate binding, phosphotyrosine residue binding, protein phosphorylated amino acid binding, phosphoprotein binding, phosphatase binding, protein kinase activity, phosphotransferase activity (alcohol group as acceptor), kinase activity, transferase activity transferring phosphorus-containing groups, and signaling receptor binding. The dominance of phosphorylation-related molecular functions underscores that hub proteins function as key regulators of kinase and phosphatase signaling networks, thereby supporting the modulation of antiviral immunity and oncogenic pathways relevant to chronic HBV infection.

Within the Pathway database menu, all available gene sets, including Gene Ontology (GO) biological process, GO cellular component, GO molecular function, and the Kyoto Encyclopaedia of Genes and Genomes (KEGG), are selected and evaluated independently. Applying the FDR cut-off (0.05), the minimum pathway size (2), and the maximum pathway size (2000), the top-most essential HBV pathways were computed. Functional enrichment analyses were performed to prioritize dominant biological pathways associated with the most topologically influential hub genes identified in Table 1 within the PN-HBV interaction network. This hub-centric enrichment strategy was employed as a mechanistic focusing approach rather than a genome-scale inferential analysis, consistent with network pharmacology frameworks that emphasize regulatory bottlenecks as key drivers of disease modulation. Enrichment platforms applied default genome-wide backgrounds, and results were interpreted descriptively to highlight convergent functional themes with established HBV-host signaling biology. Given the limited number of genes inherent to hub selection, enrichment outcomes were treated as hypothesis-generating and supportive of network topology findings rather than as statistically exhaustive pathway discovery. The results are described and illustrated in Figure 3, Figure 4, Figure 5 and Figure 6 below.

The KEGG enrichment analysis of the 10 hub genes reveals significant over-representation of multiple immune, cancer, and virus-related pathways, including the prolactin signaling pathway, pancreatic cancer, PD-L1 expression and PD-1 checkpoint pathway in cancer, EGFR-tyrosine kinase inhibitor resistance, non-small cell lung cancer, C-type lectin receptor signaling pathway, JAK-STAT signaling pathway, Kaposi sarcoma-associated herpesvirus infection, phospholipase D signaling pathway, herpes simplex virus 1 infection, hepatitis C, chemokine signaling pathway, hepatitis B, proteoglycans in cancer, and coronavirus disease (COVID-19). Notably, the majority of these enriched pathways converge on transcriptional signaling pathways controlled by EGFR, PI3K subunits, SRC-family kinases, and STATs, underscoring the significance of the hubs identified in HBV immunopathogenesis. The study’s host-directed therapeutic hypothesis is supported by the predominance of immune-oncogenic and viral infection pathways, suggesting that PN phytochemicals mainly modulate host signaling nodes that HBV exploits rather than directly inhibiting viral enzymes.

The significant enrichment of the JAK–STAT signaling pathway, prolactin signaling, and PD-1/PD-L1 checkpoint regulation is a crucial finding from these KEGG results, suggesting that the hub genes are involved in T-cell exhaustion, antiviral interferon responses, and cytokine-driven immune regulation. In order to avoid innate and adaptive immunity, HBV is known to suppress STAT1 and STAT3 phosphorylation, which leads to persistence and poor interferon responsiveness (such as decreased HBsAg clearance). Consequently, the discovery of STAT1/STAT3-centered pathways lends support to the idea that PN phytochemicals, such as rutin, procyanidin B2, and quercetin, may reverse immune exhaustion and restore antiviral signaling, in line with earlier findings that natural compounds modulate the JAK–STAT pathway in chronic HBV infection [35,36]. This hypothesis is further strengthened by the enrichment of PD-1/PD-L1 checkpoint signaling. Since PD-1-mediated T cell exhaustion is a characteristic of chronic HBV infection, substances that interact with STATs and PI3K components may secondarily modulate this checkpoint pathway and improve immune-mediated viral control.

The enrichment of EGFR-TKI resistance, pancreatic cancer, non-small cell lung cancer, and proteoglycans in cancer is indicative of shared oncogenic signaling systems engaged during hepatocarcinogenesis and chronic HBV infection rather than oncology bias in the dataset. To support cell survival, metabolic reprogramming, and malignant transformation, the HBV X protein activates the EGFR, SRC, and PI3K/AKT pathways. Inappropriate STAT3 activation is a known cause of HBV-associated hepatocellular carcinoma [25,39]. Therefore, this finding suggests that PN may not only affect viral replication but also decrease HBV-driven neoplastic development, which is supported by the overlap of hub genes with classical cancer pathways.

Moreover, a strong correlation with viral entry, replication, and antiviral immune surveillance pathways is highlighted by the enrichment of hepatitis B, hepatitis C, herpes simplex virus 1 infection, Kaposi sarcoma-associated herpesvirus infection, COVID-19, phospholipase D signaling, and the chemokine signaling pathway. Several hub genes, such as EGFR, SRC, SYK, STATs, and PI3Ks, are known host dependency factors that various viruses utilize to facilitate entry, immune evasion, and persistence. The PI3K/AKT, JAK–STAT, and chemokine pathways are where HBV, HCV, and herpesviruses converge. Recent research has shown that modifying these pathways enhances viral clearance and reduces liver inflammation [30,53]. Therefore, the KEGG analysis supports the repurposing potential of PN phytochemicals for chronic HBV therapy by providing systems-level evidence that they concurrently target antiviral immune signaling, checkpoint control, and HBV-driven oncogenic signaling. In line with previous in vitro immunomodulatory observations for PN and the network pharmacology rationale of this investigation, the convergence of various viral infection KEGG maps around the same hub genes confirms that PN likely acts as a host-directed multitarget antiviral.

Pathways such as positive regulation of smooth muscle cell proliferation, cell surface receptor protein tyrosine kinase signaling pathway, multicellular organismal-level homeostasis, enzyme-linked receptor protein signaling pathway, positive regulation of cell population proliferation, cellular response to oxygen-containing compound, response to endogenous stimulus, regulation of response to stress, cell migration, and response to oxygen-containing compound are significantly overrepresented by GO Biological Process enrichment as illustrated in Figure 4.

Growth-factor-driven receptor signaling, mediated by EGFR, SRC, PIK3CA/CB/R1, STAT1/STAT3, SYK, and PTPN11, all of which are identified as hubs in this study, is the focal point of these processes. To support hepatocyte proliferation, survival, and metabolic reprogramming, HBV utilizes receptor tyrosine kinase signaling, specifically EGFR and PI3K, while simultaneously inhibiting interferon-mediated antiviral pathways through the regulation of STAT1. Therefore, functional evidence that the hub targets identified here constitute important nodes in HBV-host interaction, rather than random protein correlations, is provided by the high enrichment of biological processes associated with receptor tyrosine kinase signaling. Systems-biology investigations of hepatocarcinogenesis and HBV development have revealed similar enrichment patterns, underscoring the significance of both processes to the consequences of chronic infection [17,20].

Further connecting the hub targets to the hepatic inflammation and fibrotic remodeling typical of chronic HBV infection is the enrichment of cell migration, proliferation, stress response regulation, and responses to oxygen-containing substances. HBV-induced liver damage is largely caused by oxidative stress reactions and redox-regulated gene expression, which also encourage neoplastic transformation and stellate cell activation. Similarly, the hepatocyte turnover and immune-cell infiltration patterns observed in chronic hepatitis are consistent with cell migration and the positive regulation of cell population proliferation, linking immunological dysregulation to the development of cirrhosis and HCC [54,55]. When considered collectively, the GO-BP analysis provides a mechanistic explanation for PN’s potential as a host-directed therapeutic agent in chronic HBV infection, supporting the interpretation that PN primarily acts through the multitarget regulation of proliferative, stress-response, and receptor-mediated signaling pathways that are dysregulated in HBV infection.

The GO Cellular Component analysis reveals a strong enrichment of phosphatidylinositol 3-kinase complex class IA, phosphatidylinositol 3-kinase complex class I, and the broader phosphatidylinositol 3-kinase complex, together with the extrinsic component of the membrane, membrane raft, membrane microdomain, transferase complex transferring phosphorus-containing groups, perinuclear region of cytoplasm, anchoring junction, and membrane protein complex, as illustrated in Figure 5.

A significant amount of the shared HBV-PN target network is physically located within class I PI3K assemblies at the cell membrane, as demonstrated by the overwhelming enrichment in PI3K complexes, which is consistent with the identification of PIK3CA, PIK3CB, and PIK3R1 as central hub proteins in our study. These complexes regulate essential processes, such as hepatocyte survival, metabolic reprogramming, and viral persistence, by acting downstream of receptor tyrosine kinases, including EGFR and SRC. The location of hub targets within PI3K complexes provides a structural explanation for how PN phytochemicals may disrupt HBV-supportive signaling scaffolds, as HBV specifically engages the PI3K/AKT axis to evade apoptosis and maintain cccDNA transcriptional activity [30,56].

Many of the identified hub proteins are involved in lipid raft-associated signaling platforms, which are known to coordinate viral entry, innate immunological sensing, and cytokine receptor signaling in hepatocytes, as indicated by the enrichment of membrane raft and membrane microdomain components. HBV internalisation and immune-evasion signalling are mediated in raft microdomains by downstream kinases such SRC, SYK, and PTPN11, as well as HBV entry cofactors like EGFR and NTCP. The hub proteins are further connected to cytoskeletal and adhesion machinery that promotes inflammation, fibrosis, and carcinogenic transformation during chronic HBV infection, facilitated by the existence of anchoring junction and membrane protein complexes [57,58]. The location of hub proteins in the perinuclear cytoplasmic area is also in line with PI3K signalling complexes and STAT1/STAT3 shuttling, which are situated close to the nucleus to control the transcription of antiviral genes [36]. Altogether, these GO cellular component findings support PN’s suggested host-directed, multitarget treatment strategy in chronic HBV infection by demonstrating that PN targets organized membrane-associated signaling complexes that HBV co-opts for survival, rather than isolated soluble enzymes.

GO Molecular Function enrichment showed significant over-representation of insulin receptor substrate binding, phosphotyrosine residue binding, protein phosphorylated amino acid binding, phosphoprotein binding, phosphatase binding, protein kinase activity, phosphotransferase activity (alcohol group as acceptor), kinase activity, transferase activity transferring phosphorus-containing groups, and signaling receptor binding, as illustrated in Figure 6.

The discovered hub proteins, which regulate phosphorylation-dependent signalling cascades, are directly mapped into these functions. To control antiviral immunity, HBV heavily depends on host tyrosine kinase and phosphatase signalling. For instance, HBx and viral envelope proteins stimulate the phosphorylation of PI3K/AKT and SRC-family kinases, while inhibiting STAT1-dependent interferon signaling through modified phosphotyrosine dynamics. The hub genes are therefore central regulators of phosphorylation signalling, which is the fundamental conceptual framework of immune activation, cytokine signalling, and viral persistence in HBV infection, rather than random interactors, as confirmed by the predominance of phosphoprotein-binding and kinase/phosphatase-associated molecular functions [36,59].

The observation that the most substantially enriched molecular function is insulin receptor substrate binding highlights the metabolic–immune interaction of HBV illness. The PI3K/AKT and EGFR pathways drive insulin signaling, hepatocyte proliferation, and survival in chronic HBV infection, which is increasingly recognized as a form of metabolic syndrome [60]. PN’s potential as a multitarget, host-directed therapeutic option in chronic HBV infection is further supported by the enrichment of kinase/phosphatase binding, phosphotyrosine recognition, and signaling-receptor binding, which collectively support a mechanistic model in which PN targets phosphorylation-dependent host signaling hubs hijacked by HBV to exert antiviral and immunomodulatory actions. Targeting host signaling nodes, such as EGFR and PI3K/AKT, is inherently complex because of their roles in normal physiology. However, PN phytochemicals generally exert partial modulatory effects rather than complete inhibition, thereby mitigating the risk of overt pathway blockade and its associated side effects. For example, EGFR functions as a host entry cofactor for HBV, and modulation of EGFR has been shown to reduce viral internalization without abolishing normal EGFR signaling. Moreover, the PI3K/AKT pathway’s regulatory interplay with HBV replication suggests that careful context-dependent modulation could shift host responses toward antiviral states without detrimental systemic inhibition. Importantly, this host-directed modulation is intended to complement rather than replace existing antiviral agents, and formal safety profiling across relevant biological systems will be essential in subsequent preclinical development [61,62].

### 2.5. Molecular Docking Analysis

Molecular docking results (kcal/mol) of the PN-HBV hub proteins, including PIK3R1, PIK3CB, STAT1, PTPN11, PIK3CA, STAT3, HCK, SYK, EGFR, and SRC, are illustrated in Table 2 below. All co-crystallized ligands were redocked into their respective protein targets using the same AutoDock Vina parameters throughout the investigation to ensure the docking protocol’s reliability and enable precise benchmarking of PN phytochemicals. After removal from crystallographic complexes, native ligands were redocked using the same grid box coordinates and exhaustiveness parameters. The full docking dataset, comprising all 27 Product Nkabinde phytochemicals against the selected hub gene targets, is provided in Supplementary Table S3. Furthermore, to assess ranking robustness, independent redocking validations were performed in Schrödinger Maestro 2022-1, in which top-ranked phytochemicals consistently retained their relative rankings; detailed results are provided in Supplementary Table S4.

In Schrodinger 2022-1, the root-mean-square deviation (RMSD) between redocked poses and their experimental crystal conformations, after protein backbone superposition, was used to assess docking pose accuracy. The visual superpositions of crystallographic and redocked ligand poses in the Supplementary Figures S12–S21 allow direct visual assessment of pose recovery in addition to numerical RMSD values. This study’s molecular docking methodology was not intended to predict absolute binding affinity, but rather to offer comparative structural insights into the multitarget interaction landscape of PN phytochemicals. As a result, protocol validation focused on experimentally grounded structural accuracy, using RMSD-based pose-recovery analysis (Supplementary Table S2) and explicit redocking of co-crystallized ligands, which verified consistent replication of known binding modes across hub targets. Supplementary Table S1 contains the full grid parameterization that guarantees the repeatability of docking searches. In the context of network pharmacology-driven target prioritization, this coupled structural validation approach facilitates the application of AutoDock Vina score for relative affinity ranking and mechanistic interpretation. Docking scores obtained from AutoDock Vina were used for comparative ranking of PN phytochemicals within the same binding pocket of each target protein, rather than as absolute predictors of binding free energy. Structural reliability of the docking workflow was ensured through explicit redocking and RMSD-based validation against crystallographic ligand conformations (Supplementary Table S2). Given the multitarget and mechanistic focus of this network pharmacology study, this combined structural validation approach was considered appropriate for prioritizing ligand–target interactions and guiding pathway-level interpretation. To ensure transparent evaluation of docking performance and ligand selectivity, binding affinity values for all PN phytochemicals and for each hub target are provided in Supplementary Table S3. These score distributions reveal consistent ranking patterns across ligands and targets, supporting the robustness of the highlighted top interactions. Several PN phytochemicals exhibit higher molecular weight and conformational flexibility relative to crystallographic reference ligands, which may influence precise pocket adaptation in rigid-receptor docking. However, the present study aimed to comparatively prioritize multitarget interactions within a network pharmacology framework rather than resolve induced-fit binding mechanisms. However, the combined interacting residues and bond types are already represented in Table 2. These results indicate that, despite structural diversity, PN phytochemicals occupy a relevant physicochemical space for protein interactions. Accordingly, docking outcomes are interpreted as indicative of relative binding propensity rather than definitive structural binding modes.

A multi-target, host-directed antiviral mechanism is strongly supported by Table 2, which demonstrates that important PN phytochemicals have binding affinities across several HBV-relevant host sites that are equal to or greater than those of the native co-crystallized ligands. The strongest effects are observed for oleanolic acid-SYK (−14.0 kcal/mol) and β-sitosterol-PIK3CB (−14.2 kcal/mol), which exhibit significantly stronger binding compared to their cognate ligands, indicating the robust stability of catalytic pockets within PI3K and SYK signaling hubs. Procyanidin B2 had increased affinity for PIK3CA and HCK, rutin bound SRC more strongly (−10.5 kcal/mol) than the reference ligand, and quercetin showed higher binding to PIK3R1 (−10.6 kcal/mol) and PTPN11 (−9.2 kcal/mol). These findings are in complete agreement with the KEGG enrichment results, which identify the PI3K/AKT, JAK–STAT, EGFR, SYK, and SRC-family pathways as key regulatory axes in cccDNA persistence, HBV immune evasion, and oncogenic development. Inhibition of these host-dependent pathways lowers HBV replication and restores immunological control. HBV is known to utilize PI3K/AKT and SRC kinases to enhance hepatocyte survival and replication, while reducing interferon responses [31,63]. Therefore, the KEGG and GO analyses in this work show pathway-level regulation, which is directly supported by the high binding of PN phytochemicals to these hubs.

Notably, the GO biological process and molecular function results highlight phosphorylation-dependent immune signaling, cytokine regulation, cell proliferation, and stress-response pathways. Procyanidin B2 docked to STAT1/STAT3 with similar affinities to their co-crystallized ligands, and strong interactions were seen for PI3K isoforms and SHP2/PTPN11. Ligands that stabilize STAT1/STAT3 and PI3K complexes may reverse immune exhaustion and improve antiviral signaling because HBV inhibits STAT1 phosphorylation to avoid interferon-mediated clearance and promotes aberrant STAT3 activation linked to fibrosis and hepatocellular cancer [64,65].

The pharmacological plausibility of the current findings is supported by the independent demonstration that natural flavonoids and triterpenoids, such as quercetin, rutin, β-sitosterol, and oleanolic acid, modulate JAK–STAT and PI3K signaling while exhibiting anti-HBV or hepatoprotective activities [7,38]. Coherent systems-level evidence that PN phytochemicals act through coordinated modulation of host signaling networks co-opted by HBV is provided by the convergence of (i) stronger-than-reference docking affinities to hub kinases and phosphatases, (ii) KEGG enrichment in HBV, JAK–STAT, PI3K/AKT, chemokine, and viral-infection pathways, and (iii) GO terms related to kinase activity, phosphotyrosine binding, membrane raft signaling, and proliferation. The proposition that PN is a promising multitarget, host-directed therapy candidate capable of preventing HBV persistence, immunological dysfunction, and disease progression is strongly supported by our findings.

Protein–ligand interaction analysis further reveals the protein active site residues involved in the binding, the hydrogen bond network, and the ionic and hydrophobic interactions responsible for the complex stability are illustrated in Table 3.

While molecular docking revealed multiple PN phytochemicals with significant binding affinities to essential hub targets, pharmacokinetic properties are a crucial factor in therapeutic application, especially for structurally intricate natural products that may face challenges in solubility, permeability, or metabolic stability. Contemporary computational drug development methodologies increasingly use in silico absorption, distribution, metabolism, excretion, and toxicity (ADMET) profiling to enhance target-based screening of the 27 PN phytochemicals in Supplementary Figure S5. Tools like SwissADME facilitate rapid forecasting of gastrointestinal absorption, lipophilicity, aqueous solubility, blood–brain barrier permeability, cytochrome P450 interactions, and adherence to recognized drug-likeness standards, making them essential for the preliminary assessment of natural products [89]. A study by Ghani et al. [90] reported that integrating ADMET filters with docking and network pharmacology significantly improves the discovery of bioactive phytochemicals with enhanced translational potential. PN is a polyherbal formulation in which the constituent phytochemicals may produce synergistic pharmacokinetic effects, such as enhanced solubility, modulation of metabolic enzymes, and improved intestinal permeability, effects increasingly acknowledged in the field of herbal systems pharmacology. While such formulation-level interactions may partially mitigate individual compound limitations, rigorous computational and experimental pharmacokinetic validation remains essential. Accordingly, future investigations will incorporate comprehensive ADMET and drug-likeness profiling to further refine lead phytochemicals emerging from the present mechanistic screening framework [90].

Moreover, molecular docking provides quantitative estimates of ligand–protein binding affinity and identifies potential interaction modes within catalytic or regulatory pockets; it does not directly predict functional efficacy or pharmacodynamic directionality (agonism versus antagonism). Accordingly, the docking results in this study are interpreted as indicators of relative binding propensity and target engagement rather than definitive measures of biological effect. Functional implications are therefore inferred based on the established mechanistic roles of each hub protein in HBV pathogenesis. Importantly, all major hub targets identified in this work, PIK3CA, PIK3CB, PIK3R1, SRC, HCK, SYK, EGFR, STAT3, and PTPN11, are host signaling proteins that HBV actively hijacks to promote viral replication, immune suppression, hepatocyte survival, and oncogenic progression. Extensive literature demonstrates that activation of PI3K/AKT signaling, SRC-family kinases, EGFR pathways, SYK-mediated immune dysregulation, aberrant STAT3 signaling, and SHP2/PTPN11-driven growth factor signaling supports HBV persistence and hepatocellular carcinoma development. Consequently, therapeutic strategies that inhibit these host pathways consistently reduce HBV replication, restore antiviral immune signaling, and limit disease progression. Following the present docking analysis targeted ATP-binding clefts and catalytic pockets of kinases and phosphatases, the observed high-affinity binding of PN phytochemicals most plausibly reflects competitive inhibition, consistent with classical small-molecule kinase inhibitor mechanisms. In contrast, STAT1, an antiviral transcription factor suppressed by HBV, displayed comparable ligand engagement rather than strong displacement, aligning with the concept of stabilizing or restoring antiviral signaling rather than inhibiting it. Therefore, within a host-directed therapeutic framework, strong docking affinities toward PI3K isoforms, SRC-family kinases, EGFR, SYK, STAT3, and PTPN11 are mechanistically consistent with inhibitory modulation beneficial for HBV control, while engagement of STAT1 aligns with restoration of interferon-mediated antiviral activity. Molecular docking results reflect predicted binding affinity and spatial compatibility between phytochemicals and target proteins, but do not establish functional efficacy or directionality of modulation. For kinase-regulated transcription factors such as STAT1 and STAT3, ligand binding within regulatory pockets previously associated with phosphorylation and dimerization interfaces has been widely linked to pathway inhibition in experimental systems. Accordingly, interactions observed here are discussed as putative inhibitory hypotheses rather than confirmed functional outcomes. Definitive conclusions regarding activation or suppression require biochemical and cellular validation. Nonetheless, we emphasize that functional outcomes cannot be inferred from docking alone and require experimental validation in cellular and in vivo HBV models [91,92]. All network associations and docking-derived affinities reported herein represent computational predictions intended to prioritize candidate targets and phytochemicals for future experimental validation, rather than evidence of direct functional modulation or therapeutic efficacy.

## 3. Materials and Methods

### 3.1. Compound Screening and Preparation

SMILES of the 27 phytochemicals of PN were retrieved from PubChem. The Swiss Target Prediction database was used to computationally predict which proteins these inhibitors will target [93,94]. A total of 1034 genes were retrieved and saved in an Excel spreadsheet. The gene dataset was cleaned, and 390 genes were used for further investigation. GeneCards, a database containing information on all gene sets related to diseases, was used to predict and identify human proteins associated with HBV [95]. A total of 12,885 proteins (genes) were collected, cleaned, and then stored in an Excel spreadsheet for additional screening on the VENNY website. The computation of common proteins in VENNY 2.1.0 yielded a Venn diagram showing the intersecting proteins for PN phytochemicals and HBV [96]. To reduce false-positive associations inherent to ligand-based target prediction, SwissTargetPrediction outputs were filtered by predicted probability and restricted to human protein targets. Predicted targets were subsequently intersected with a curated set of HBV-associated host genes (Table 1) to retain only disease-relevant proteins. Network topology screening was then applied after STRING-based network construction to prioritize highly connected, centrally positioned targets, thereby further minimizing spurious predictions. This multi-layer filtering framework ensured biologically meaningful and mechanistically robust target selection.

### 3.2. Interaction Network and Hub Genes Analysis

Following the examination of common proteins, the interaction network was constructed using the Search Tool for the Retrieval of Interacting Genes (STRING) database. For further investigation, the interaction network reveals interactions with a high degree of confidence [97]. The interaction network constructed using STRING represents a functional association network integrating experimentally supported interactions, curated pathway relationships, and co-expression evidence rather than exclusively direct physical protein–protein binding interfaces. Accordingly, network proximity reflects biological connectivity and shared functional involvement in cellular processes rather than structural interaction surfaces relevant to direct protein–protein targeting. The common genes were uploaded, and the interaction network was computed after selecting Homo sapiens from the drop-down menu and selecting the multiple proteins icon in the STRING database. The examined interaction network was exported and stored in high-resolution Portable Network Graphics (PNG) format. To identify hub genes, the network was also uploaded to Cytoscape [98]. The hub genes, which are involved in key biological processes and ensure the integrity of the interaction network, are often referred to as important genes. They disclose the mechanistic dynamics of illnesses by influencing cell functioning. The latest version of Cytoscape, 3.10.3, was downloaded. Cytohubba and yfile were then included in the program. SwissTargetPrediction was used to identify putative protein targets of the PN phytochemicals, retaining only targets with a prediction probability ≥0.1 to ensure moderate-to-high confidence interactions. Predicted targets were intersected with hepatitis B virus-related genes obtained from curated disease databases, yielding 359 overlapping PN-HBV-associated targets for subsequent analysis. Protein–protein interaction (PPI) networks were constructed using the STRING database with a high-confidence interaction score threshold of ≥0.7. Only experimentally validated interactions, curated pathway database evidence, and co-expression channels were included, while text-mining-only associations were excluded to minimize spurious functional links. The resulting interaction network was imported into Cytoscape for topological analysis, and hub genes were identified based on degree centrality and network connectivity metrics. These thresholds were selected in accordance with established network pharmacology practices to ensure robustness and biological relevance of key target identification.

### 3.3. Functional Enrichment Analysis

The ShinyGO 0.85 database was used for Kyoto Encyclopedia of Genes and Genomes (KEGG) and Gene Ontology (GO) pathway analysis [99]. To understand their biological roles, identify potential treatment targets, and elucidate the mechanisms behind HBV, the hub genes were added to the database, and their functional enrichment was assessed [100,101]. The Benjamini–Hochberg method and the hypergeometric test are used to calculate *p*-values and False Discovery Rates (FDR) [102]. Fold enrichment is calculated by dividing the percentage of genes in a pathway among the ten hub genes by the equivalent percentage of background genes. The effect magnitude is directly measured by the fold enrichment, and the statistical significance is indicated by the FDR [103]. This study employed an FDR cut-off of 0.05 to show the 10 most enriched pathways. The average sorting of the pathways is done using FDR and fold enrichment when ‘Select by FDR and Sort by Enrichment’ was selected.

### 3.4. Systems Preparation, Molecular Docking, and Protein–Ligand Interaction Analysis

To predict and assess binding energies for the PN-HBV hub protein complexes, molecular docking was performed using PyRx. The complexes’ putative multi-targeted molecular activities, which consistently match the network pharmacology structure, are revealed by molecular docking [104]. Moreover, many therapeutic targeted networks, rather than single-targeted ones, are revealed by molecular docking. Molecular docking was performed using AutoDock Vina implemented within the PyRx virtual screening platform. The PubChem database was used to obtain the three-dimensional structures of the PN phytochemicals. These structures were then loaded into PyRx, where ligand geometry optimization and energy minimization were carried out using Open Babel with the universal force field (UFF). Prior to docking and optimization, water molecules and co-crystallized ligands were eliminated from the 10 target protein structures that were acquired from the Protein Data Bank (PDB) (PTPN11 PDB ID 6BN5, SRC PDB ID 2SRC, STAT1 PDB ID 1YVL, STAT3 PDB ID 6NUQ, SYK PDB ID 4XG4, PIK3CA PDB ID 5SXA, PIK3CB PDB ID 4PUZ, PIK3R1 PDB ID 5XGI, EGFR PDB ID 4R3P, and HCK PDB ID 5H0B). The Vina Wizard was used to construct docking grids that included the active or regulatory binding domains of each target protein. Molecular docking was performed using AutoDock Vina (v1.1.2) within PyRx 0.8, following a standardized, reproducible protocol. Protein structures were prepared by removing co-crystallized ligands and water molecules, followed by the addition of polar hydrogens and Gasteiger charges, with receptors treated as rigid. Ligands were energy-minimized using Open Babel (UFF) and docked with full torsional flexibility. Docking grids were centered on the crystallographic binding sites and fully covered the active or regulatory pockets, with grid box dimensions shown in Supplementary Table S1. Docking was performed with an exhaustiveness value of 8, yielding 9 poses per ligand. The pose with the lowest predicted binding free energy and correct pocket occupation was selected for analysis. Redocking validation was performed using the Schrödinger Suite (Maestro v2022-1) to assess the reliability of the docking protocol. For each hub protein, the co-crystallized ligand was extracted from the experimental structure, and the receptor was prepared using the Protein Preparation Wizard with standard settings, including bond-order assignment, hydrogen addition, hydrogen-bond network optimization, and restrained energy minimization. The extracted native ligand was then re-docked into the original binding site using Glide under the same docking parameters applied in the main docking experiments. The resulting docked pose was superimposed onto the crystallographic ligand conformation, and root-mean-square deviation (RMSD) values were calculated over heavy atoms to evaluate pose recovery. The reproduced poses showed acceptable agreement with the experimental conformations, supporting the robustness and reproducibility of the docking workflow. The binding affinities were reported as binding free energy values (kcal/mol) after molecular docking with AutoDock Vina using default exhaustiveness parameters. To examine hydrogen bonding, hydrophobic, and π-alkyl interactions, docking postures were selected based on the lowest binding energy [104,105]. Protein–ligand interactions computed with BIOVIA Discovery Studio software v21.1.0.20298 reveal the protein active site residues involved in binding, the hydrogen bond network, and the ionic and hydrophobic interactions responsible for a stable and specific protein–ligand complex, highlighting the synergistic roles in inhibiting HBV progression [106,107].

## 4. Conclusions

Product Nkabinde (PN) has a great potential to be repurposed as a host-directed treatment option for chronic hepatitis B virus (HBV) infection, according to convergent systems-level data presented in this work. PN is an experimentally validated African polyherbal formulation with demonstrated antiviral and immunomodulatory activity in the context of HIV infection and is currently undergoing preclinical development for HIV-related therapeutic applications. This present study does not introduce PN as a newly discovered antiviral agent but rather repurposes this clinically characterized formulation for hepatitis B virus (HBV) through a host-directed network pharmacology framework. Unlike prior reports on polyherbal interventions for HBV, which predominantly emphasize empirical extract-level antiviral screening or hepatoprotective effects, this work systematically interrogates the mechanistic convergence between PN phytochemicals and HBV-associated host dependency pathways. By integrating 27 structurally defined PN constituents with HBV-related targets, we identify a focused host interaction network and prioritize key regulatory hub genes governing immune signaling, inflammatory modulation, and viral persistence. Subsequent structure-based molecular docking further provides quantitative support for multitarget host protein engagement, linking PN’s established immunomodulatory properties to molecular nodes central to HBV pathobiology. This integrative repurposing strategy offers a mechanistically grounded perspective on polyherbal antiviral therapy and represents a distinct advancement beyond descriptive phytotherapy studies in HBV research. Instead of relying on single-enzyme viral suppression, we demonstrate that the primary bioactive phytochemicals in PN simultaneously engage numerous HBV-relevant host targets by combining network pharmacology, multi-omics pathway enrichment, and molecular docking. Key signaling hubs that control interferon responses, T-cell fatigue, hepatocyte survival, inflammatory signaling, and oncogenic transformation, such as STAT1, STAT3, EGFR, SRC, SYK, PIK3CA, PIK3CB, PIK3R1, HCK, and PTPN11, exhibited strong binding affinities for PN compounds. In addition to GO biological processes connected with cytokine signaling, kinase/phosphatase activity, stress response, cellular proliferation, and membrane-associated viral entry complexes, these same proteins were prominent in KEGG antiviral, immunological, and cancer-driven pathways. PN phytochemicals, such as β-sitosterol, oleanolic acid, quercetin, rutin, and procyanidin B2, showed equal or greater affinity than native co-crystallized ligands across the identified hubs, suggesting a plausible capacity to competitively modulate host signaling that HBV hijacks for persistence and immune evasion. Molecular docking further confirmed functional relevance. When combined, these results provide compelling evidence for the main focus of this work: Instead of acting as a single-target antiviral, PN functions as a multitarget, network-level modulator of host pathways necessary for HBV survival, making it a viable option for repurposing within HBV functional-cure regimens. Such a host-directed strategy is directly in line with current therapy paradigms that aim to prevent immune exhaustion, inhibit the transcriptional support of cccDNA, restore interferon responsiveness, and prevent the development of fibrosis and hepatocellular carcinoma. The overlap of immunomodulatory, anti-oncogenic, and antiviral markers suggests that PN may be able to address the carcinogenic risk and chronic inflammation that characterize the long-term HBV disease burden, in addition to viral replication. Although PN is a polyherbal formulation, this study employed a compound-resolved network pharmacology strategy in which individually characterized phytochemicals were modeled against host HBV-relevant targets. This approach ensures mechanistic reproducibility independent of formulation batch variability by anchoring analyses to fixed molecular structures. Contemporary polyherbal drug development increasingly adopts marker-compound-based standardization frameworks, in which key bioactive constituents serve as quality-control surrogates to ensure compositional consistency across production batches. The high-affinity and network-central phytochemicals identified here, therefore, not only underpin the mechanistic basis of PN’s host-directed effects but also provide rational candidates for future standardization and translational quality assurance. The findings in this study provide a strong molecular explanation for PN as an African polyherbal formulation relevant to public health, with convincing scientific support for repurposing in HBV therapy. However, experimental validation and controlled clinical assessment remain the next crucial steps. Thus, this work provides a data-driven platform for advancing economical, multi-mechanistic treatments in HBV-endemic areas, thereby bridging the gap between traditional medicine and contemporary drug discovery. Comprehensive in vitro and in vivo trials, including evaluation of PN’s effects on HBV replication, cccDNA activity, immunological reconstitution, and fibrosis regression, should be used in future research to validate these in silico predictions. Furthermore, to assess the safety, pharmacokinetics, and therapeutic efficacy of PN as an adjuvant or stand-alone host-directed therapy for chronic HBV infection, carefully planned clinical trials are advised.

## Figures and Tables

**Table 1:** PN-HBV Intercepting Hub Genes with Clinical Relevance.

Common Name	Full Name	Function	Association with HBV	Clinical Relevance	References
SRC	Proto-Oncogene Tyrosine-Protein Kinase Src	Non-receptor tyrosine kinase regulating signaling pathways involved in adhesion, proliferation, survival, and immune signaling	HBV manipulates Src-family kinases to enhance entry, replication, and immune evasion	Activation of SRC correlates with hepatocyte proliferation, progression to cirrhosis, and HCC development in chronic HBV patients	[23,24,25]
EGFR	Epidermal Growth Factor Receptor	Receptor tyrosine kinase regulating growth, survival, and differentiation	HBV activates EGFR signaling to support replication and hepatocyte survival	EGFR upregulation is associated with fibrosis progression and HCC risk in HBV-infected cohorts; EGFR inhibitors reduce HBV DNA levels in experimental models	[26,27,28,29]
PIK3R1	Phosphoinositide-3-Kinase Regulatory Subunit 1	Regulates PI3K catalytic activity and downstream AKT signaling	HBV activates PI3K–AKT signaling to suppress apoptosis	PI3K pathway activation is associated with poorer antiviral treatment response and higher HBV viral load	[29,30,31]
PIK3CB	Phosphoinositide-3-Kinase Catalytic Subunit Beta	Catalytic subunit mediating AKT phosphorylation and metabolic control	Supports HBV replication and hepatocyte metabolic reprogramming	PI3Kβ activation links to liver fibrosis severity and inflammation markers in chronic HBV patients	[32,33,34]
STAT1	Signal Transducer and Activator of Transcription 1	Key mediator of type I/II interferon signaling and antiviral response	HBV suppresses STAT1 phosphorylation to evade immunity	Reduced STAT1 signaling is associated with poor interferon therapy response and persistent HBV DNA positivity	[35,36,37]
STAT3	Signal Transducer and Activator of Transcription 3	Cytokine-responsive transcription factor regulating proliferation and inflammation	HBV induces aberrant STAT3 activity promoting immune tolerance and oncogenesis	Constitutive STAT3 activation correlates with HCC risk, higher HBV load, and inflammatory cytokine signatures	[38,39,40]
SYK	Spleen Tyrosine Kinase	Non-receptor kinase mediating innate and adaptive immune signaling	HBV alters SYK signaling to dysregulate B-cell and innate responses	Clinical samples show SYK dysregulation associated with chronic HBV immune dysfunction and impaired seroconversion	[41,42,43]
HCK	Hematopoietic Cell Kinase	Src-family kinase regulating macrophage and neutrophil signaling	HBV utilizes HCK to suppress antiviral cytokine production	Elevated HCK expression in HBV patients correlates with immune suppression and HBV chronicity	[44,45,46]
PTPN11	Protein Tyrosine Phosphatase Non-Receptor Type 11 (SHP2)	Phosphatase linking growth factor receptors to MAPK and PI3K pathways	HBV activates PTPN11/SHP2 to promote cell survival and viral replication	High PTPN11 expression in HBV livers correlates with fibrosis progression and HCC development	[47,48,49]
PIK3CA	Phosphatidylinositol-4,5-Bisphosphate 3-Kinase Catalytic Subunit Alpha	Catalytic subunit driving PI3K–AKT–mTOR signaling	HBV activates PIK3CA to inhibit apoptosis and sustain replication	PIK3CA mutations and activation are enriched in HBV-associated HCC and predict poor prognosis	[50,51,52]

**Table 2:** Binding affinities (kcal/mol) of PN phytochemicals docked against hub target proteins compared with their co-crystallized ligands.

Target Protein	Co-Crystallized Ligand (kcal/mol)	Phytochemical	Binding Energy (kcal/mol)	Comparative Outcome
STAT1	−10.8	Procyanidin B2	−9.0	Slightly weaker but significant binding
STAT3	−8.9	Procyanidin B2	−8.7	Comparable binding
PIK3CB	−7.9	β-Sitosterol	−14.2	Much stronger binding
SYK	−8.3	Oleanolic acid	−14.0	Much stronger binding
PIK3CA	−7.3	Procyanidin B2	−9.5	Stronger binding
PIK3R1	−8.2	Quercetin	−10.6	Stronger binding
EGFR	−8.4	Diosgenin	−9.4	Stronger binding
SRC	−7.8	Rutin	−10.5	Stronger binding
HCK	−7.9	Procyanidin B2	−10.5	Stronger binding
PTPN11	−7.5	Quercetin	−9.2	Stronger binding

**Table 3:** Detailed 2D Protein–Ligand Interaction Analysis of PN Phytochemicals with HBV Hub Proteins (Also represented in Supplementary Figures S1–S10).

Protein	Ligand	Combined Interacting Residues & Bond Types	Comparison with Co-Crystallized Ligand Binding Mode	Implications in HBV Pathogenesis	References
HBV Hub Proteins (Also represented in Supplementary Figures S1–S5)					
STAT1	Procyanidin B2	H-bonds: Glu1605, Arg1602, Ser1604, Ser1606, Lys1584, Glu1563, Lys1636, Ser1560, Arg1608; vdW: Glu1632, Tyr1634, Tyr1651, Val1653, Leu1556; Hydrophobic: Val1653, Leu1556	Docked ligand forms more hydrogen bonds and stronger electrostatic interactions compared with the co-crystal, improving binding affinity.	Enhances IFN-mediated antiviral signaling, reducing HBV transcription and replication.	[64,66,67]
STAT3	Procyanidin B2	H-bonds: Lys233, Asp237, Thr346, Ile258, Arg325, Asn315; Electrostatic: Glu229; vdW: Gln232, Ser319, Lys244; π-alkyl: Ala250	Docked ligand shows extra hydrogen bonds and π-interactions not present in the co-crystal, indicating tighter ATP-pocket binding.	Inhibits STAT3 oncogenic signaling activated in HBV and HCC.	[68,69,70,71]
PTPN11	Quercetin	π-cation: Arg111; H-bonds: Thr218, Thr219; π-alkyl: Lys492, Pro491; vdW: Met496, Gln495, Leu254, Glu250	Docked ligand retains Arg111 π-cation and adds new hydrogen bonds, leading to stronger catalytic pocket occupation.	Blocks PTPN11-mediated RAS/MAPK signaling involved in HBV survival and tumorigenesis.	[71,72,73]
HCK	Procyanidin B2	H-bonds: Ser345, Asp348, Ala293, Thr338, Glu339, Met341; π–alkyl/π–π: Phe405, Val281, Leu393; Electrostatic: Lys295, Asp404	Docked ligand creates more polar and charged interactions than the co-crystal, suggesting higher inhibitory potential.	Targets Src-family kinase supporting HBV replication signaling.	[74,75,76]
SRC	Rutin	H-bonds: Asp198, Thr199, Glu160, Arg161, Val200, His202; π–π: Tyr320; vdW: Ala165, Pro166	Docked ligand displays extensive hinge-region hydrogen bonding and π–π stacking stronger than the co-crystal.	Suppresses HBV-associated proliferation and immune evasion pathways.	[77,78,79]
EGFR	Diosgenin	H-bonds: Arg841, Asp837, Thr338, Thr403; π-cation: Lys879; hydrophobic: Ile878, Ala920, Pro919	Docked ligand shows more direct H-bonds and hydrophobic packing than water-mediated co-crystal interactions.	Disrupts EGFR signaling, enhancing HBV entry and disease progression.	[80,81,82]
PIK3R1	Quercetin	H-bonds: Cys838, Ser629, Asp626, Gln630, Lys271; π-π: Phe666; vdW: His670, Asn756	Docked ligand retains π-π stacking and adds multiple H-bonds, improving regulatory interface engagement compared to the co-crystal.	Blocks the PI3K/AKT cell survival pathway exploited by HBV.	[83,84,85]
PIK3CA	Procyanidin	H-bonds: Gln682, Asp133, Thr462, Asn428; π-anion: Glu135; hydrophobic: Val461, Leu645	Docked ligand reinforces catalytic Glu135 contact and adds new hydrogen bonds, suggesting stronger inhibition than the co-crystal.	Inhibits the PI3K catalytic subunit, limiting HBV replication and HCC oncogenesis.	[86,87]
SYK	Oleanolic acid	H-bonds: Glu452, Asp512; Alkyl/π-alkyl: Leu377, Val385, Pro455, Met450; vdW contacts abundant	Docked ligand maintains catalytic pocket contacts and increases hydrophobic packing relative to the co-crystal.	Modulates SYK-mediated immune signaling relevant to HBV inflammation.	[87,88]
PIK3CB	β-sitosterol	H-bonds: Asp512, Glu452; Alkyl: Val385, Leu377, Pro455, Met448; vdW extensive contacts	Docked ligand demonstrates stronger hydrophobic pocket filling than the co-crystal despite limited hydrogen bonding.	PIK3CB involvement in AKT signaling contributes to HBV persistence; inhibition is beneficial.	[86,87]

## Data Availability

The data are available upon request.

## Supplementary Materials

The following supporting information can be downloaded at: https://www.mdpi.com/article/10.3390/ph19040627/s1, Table S1: Docking Grid Parameters, Table S2: RMSD redocking validation results of co-crystallized ligands, Table S3: All the docking results, Table S4: Redocked Results, Table S5: In silico ADMET and drug-likeness profiling of Product Nkabinde phytochemicals (SwissADME), Figures S1–S10: 2D diagram of Protein-ligand interactions of the 10 hub genes. Figure S11: Interaction Network of the 359 PN-HBV common genes. Figures S12–S21: The original cocrystallized ligand is in green, while the redocked cocrystallized ligand is in magenta.

## References

[1] Rajbhandari R., Nguyen V.H., Knoble A., Fricker G., Chung R.T. (2025). Advances in the Management of Hepatitis B. BMJ.

[2] Shechter O., Sausen D.G., Dahari H., Vaillant A., Cotler S.J., Borenstein R. (2025). Functional Cure for Hepatitis B Virus: Challenges and Achievements. Int. J. Mol. Sci..

[3] Hui R.W.H., Mak L.Y., Fung J., Seto W.K., Yuen M.F. (2025). Prospect of Emerging Treatments for Hepatitis B Virus Functional Cure. Clin. Mol. Hepatol..

[4] Ugbaja S.C., Omerigwe S.A., Ndlovu S.M.Z., Ngcobo M., Gqaleni N. (2025). Evaluating the Efficacy of Repurposed Antiretrovirals in Hepatitis B Virus Treatment: A Narrative Review of the Pros and Cons. Int. J. Mol. Sci..

[5] Chen J., Ji D., Jia J., Zhuang H., Zhang X., Wang F.-S., Zhang W., Dou X., Tanwandee T., Sarin S.K. (2025). Functional Cure with New Antiviral Therapy for Hepatitis B Virus: A Systematic Review and Meta-Analysis. Hepatol. Int..

[6] Badia R., Garcia-Vidal E., Ballana E. (2022). Viral-Host Dependency Factors as Therapeutic Targets to Overcome Antiviral Drug-Resistance: A Focus on Innate Immune Modulation. Front. Virol..

[7] Alsaikhan F., Farhood B. (2025). Phytochemical-Based Immunomodulation: A Promising Therapeutic Approach for Viral Infections. Phyther. Res..

[8] Mngomezulu K., Madlala P., Nkabinde S.A., Nkabinde M., Ngcobo M., Gqaleni N. (2025). Herbal Formulations, Product Nkabinde and Gnidia Sericocephala, Exhibit Potent in Vitro Activity against HIV-1 Infection. Front. Pharmacol..

[9] Setlhare B., Letsoalo M., Nkabinde S.A., Nkabinde M., Mzobe G., Mtshali A., Parveen S., Ngcobo S., Invernizzi L., Maharaj V. (2024). An in Vitro Study to Elucidate the Effects of Product Nkabinde on Immune Response in Peripheral Blood Mononuclear Cells of Healthy Donors. Front. Pharmacol..

[10] Yu Y., Wang Z., Yang A., Wang Y., Bao C., Zhuo L., Han Q., Zhao H., Zhang J. (2025). Chronic HBV Infection Impairs the Glucose Metabolism and Effector Function of NK Cells via HBsAg/IL-15/MTOR Axis. Cell Death Dis..

[11] Li L., Kar S. (2025). Leveraging Network Pharmacology for Drug Discovery: Integrative Approaches and Emerging Insights. Med. Drug Discov..

[12] Tembeni B., Sciorillo A., Invernizzi L., Klimkait T., Urda L., Moyo P., Naidoo-Maharaj D., Levitties N., Gyampoh K., Zu G. (2022). HPLC-Based Purification and Isolation of Potent Anti-HIV and Latency Reversing Daphnane Diterpenes from the Medicinal Plant Gnidia Sericocephala. Viruses.

[13] Maharaj V., Dashnie N., Tembeni B. (2022). Characterization and Isolation of Anti-HIV and Latency Reversal Agents for the Development of a Herbal Mixture of Gnidia Sericocephala, Senna Italica, Sclerocarya.

[14] Ugbaja S.C., Ngcobo M., Nkabinde S.A., Nkabinde M., Gqaleni N. (2026). Molecular Investigation of Product Nkabinde in HIV Therapy: A Network Pharmacology and Molecular Docking Approach. Int. J. Mol. Sci..

[15] Frericks N., Klöhn M., Lange F., Pottkämper L., Carpentier A., Steinmann E. (2025). Host-Targeting Antivirals for Chronic Viral Infections of the Liver. Antiviral Res..

[16] Parvez M.K., Arbab A.H., Al-Dosari M.S., Al-Rehaily A.J. (2016). Antiviral Natural Products Against Chronic Hepatitis B: Recent Developments. Curr. Pharm. Des..

[17] Kumar S. (2021). Protein–Protein Interaction Network for the Identification of New Targets Against Novel Coronavirus. Silico Modeling of Drugs Against Coronaviruses. Methods in Pharmacology and Toxicology.

[18] Song X., Zhu J., Sun F., Wang N., Qiu X., Zhu Q., Qi J., Wang X. (2024). Target-Centric Analysis of Hepatitis B: Identifying Key Molecules and Pathways for Treatment. Sci. Rep..

[19] Loscalzo J. (2022). Molecular Interaction Networks and Drug Development: Novel Approach to Drug Target Identification and Drug Repositioning. FASEB J..

[20] Zheng S., Qi W., Xue T., Zao X., Xie J., Zhang P., Li X., Ye Y., Liu A. (2024). Chinese Medicine in the Treatment of Chronic Hepatitis B: The Mechanisms of Signal Pathway Regulation. Heliyon.

[21] Nevola R., Beccia D., Rosato V., Ruocco R., Mastrocinque D., Villani A., Perillo P., Imbriani S., Delle Femine A., Criscuolo L. (2023). HBV Infection and Host Interactions: The Role in Viral Persistence and Oncogenesis. Int. J. Mol. Sci..

[22] Ajoyan H., Douglas M.W., Tu T. (2023). Targeting liver metabolism: A pathway to cure hepatitis B virus?. Expert Rev. Gastroenterol. Hepatol..

[23] Klein N.P., Bouchard M.J., Wang L.H., Kobarg C., Schneider R.J. (1999). Src Kinases Involved in Hepatitis B Virus Replication. EMBO J..

[24] Lu J.W., Yang W.Y., Tsai S.M., Lin Y.M., Chang P.H., Chen J.R., Wang H.D., Wu J.L., Jin S.L.C., Yuh C.H. (2013). Liver-Specific Expressions of HBx and Src in the P53 Mutant Trigger Hepatocarcinogenesis in Zebrafish. PLoS ONE.

[25] Sivasudhan E., Blake N., Lu Z., Meng J., Rong R. (2022). Hepatitis B Viral Protein HBx and the Molecular Mechanisms Modulating the Hallmarks of Hepatocellular Carcinoma: A Comprehensive Review. Cells.

[26] Gan C.J., Li W.F., Li C.N., Li L.L., Zhou W.Y., Peng X.M. (2020). EGF receptor inhibitors comprehensively suppress hepatitis B virus by downregulation of STAT3 phosphorylation. Biochem. Biophys. Rep..

[27] Chen S.W., Himeno M., Koui Y., Sugiyama M., Nishitsuji H., Mizokami M., Shimotohno K., Miyajima A., Kido T. (2020). Modulation of Hepatitis B Virus Infection by Epidermal Growth Factor Secreted from Liver Sinusoidal Endothelial Cells. Sci. Rep..

[28] Herrscher C., Roingeard P., Blanchard E. (2020). Hepatitis B Virus Entry into Cells. Cells.

[29] Yu J., Shen Z., Chen S., Liu H., Du Z., Mao R., Wang J., Zhang Y., Zhu H., Yang S. (2023). Inhibition of HBV replication by EVA1A via enhancing cellular degradation of HBV components and its potential therapeutic application. Antivir. Res..

[30] Jose-Abrego A., Roman S., Laguna-Meraz S., Panduro A. (2023). Host and HBV Interactions and Their Potential Impact on Clinical Outcomes. Pathogens.

[31] Ijspeert H., Dalm V.A.S.H., Van Zelm M.C., Edwards E.S.J. (2024). Hyperactivation of the PI3K Pathway in Inborn Errors of Immunity: Current Understanding and Therapeutic Perspectives. Immunother. Adv..

[32] He Y., Sun M.M., Zhang G.G., Yang J., Chen K.S., Xu W.W., Li B. (2021). Targeting PI3K/Akt Signal Transduction for Cancer Therapy. Signal Transduct. Target. Ther..

[33] Tian L.Y., Smit D.J., Jücker M. (2023). The Role of PI3K/AKT/MTOR Signaling in Hepatocellular Carcinoma Metabolism. Int. J. Mol. Sci..

[34] Yang Y., Jia X., Qu M., Yang X., Fang Y., Ying X., Zhang M., Wei J., Pan Y. (2023). Exploring the Potential of Treating Chronic Liver Disease Targeting the PI3K/Akt Pathway and Polarization Mechanism of Macrophages. Heliyon.

[35] Tolomeo M., Cavalli A., Cascio A. (2022). STAT1 and Its Crucial Role in the Control of Viral Infections. Int. J. Mol. Sci..

[36] Lei Z., Wang L., Gao H., Guo S., Kang X., Yuan J., Lv Z., Jiang Y., Yi J., Chen Z. (2024). Mechanisms Underlying the Compromised Clinical Efficacy of Interferon in Clearing HBV. Virol. J..

[37] Xu B., Tang B., Wei J. (2021). Role of STAT1 in the Resistance of HBV to IFN-α. Exp. Ther. Med..

[38] Patel B., Zheng X., Kahn L.M., Schneider S.M., Pineda J.E., Riba M.N., Ouchen K., Meril S., Nash A.P., Wang J. (2025). STAT3 Mediates an Inflammation-Induced Microbial Defense Response and Regulates Pathogen Control and Clearance by Macrophages. J. Immunol..

[39] Zhao J., Qi Y.-F., Yu Y.-R. (2021). STAT3: A key regulator in liver fibrosis. Ann. Hepatol..

[40] Yang Y., Zheng B., Han Q., Zhang C., Tian Z., Zhang J. (2016). Targeting Blockage of STAT3 Inhibits Hepatitis B Virus-Related Hepatocellular Carcinoma. Cancer Biol. Ther..

[41] Theobald S.J., Simonis A., Mudler J.M., Göbel U., Acton R., Kohlhas V., Albert M., Hellmann A., Malin J.J., Winter S. (2022). Spleen Tyrosine Kinase Mediates Innate and Adaptive Immune Crosstalk in SARS-CoV-2 MRNA Vaccination. EMBO Mol. Med..

[42] Edwards E.S.J., Chatelier J., Snell G.I., Seo G.H., Khang R., O’Hehir R.E., Bosco J.J., van Zelm M.C. (2025). Novel SYK Variant Causes Enhanced SYK Autophosphorylation and PI3K Activation in an Antibody-Deficient Patient. J. Clin. Immunol..

[43] Yu F., Zhu Y., Li S., Hao L., Li N., Ye F., Jiang Z., Hu X. (2024). Dysfunction and Regulatory Interplay of T and B Cells in Chronic Hepatitis B: Immunotherapy and Emerging Antiviral Strategies. Front. Cell. Infect. Microbiol..

[44] Shu S.T., Chen L., Gonzalez-Areizaga G., Smithgall T.E. (2024). Constitutive Activation of the Src-Family Kinases Fgr and Hck Enhances the Tumor Burden of Acute Myeloid Leukemia Cells in Immunocompromised Mice. Sci. Rep..

[45] Xia Y., Protzer U. (2017). Control of Hepatitis B Virus by Cytokines. Viruses.

[46] Poh A.R., O’Donoghue R.J.J., Ernst M. (2015). Hematopoietic Cell Kinase (HCK) as a Therapeutic Target in Immune and Cancer Cells. Oncotarget.

[47] Fadhal E. (2025). Network Centric Identification of PI3K/Akt Hub Proteins as Key Oncogenic Drivers and Therapeutic Targets. Sci. Rep..

[48] Wang Q., Pan W., Wang S., Pan C., Ning H., Huang S., Chiu S.-H., Chen J.-L. (2021). Protein Tyrosine Phosphatase SHP2 Suppresses Host Innate Immunity against Influenza A Virus by Regulating EGFR-Mediated Signaling. J. Virol..

[49] Bard-Chapeau E.A., Li S., Ding J., Zhang S.S., Zhu H.H., Princen F., Fang D.D., Han T., Bailly-Maitre B., Poli V. (2011). PTPN11/Shp2 Acts as a Tumor Suppressor in Hepatocellular Carcinogenesis. Cancer Cell.

[50] Zhang H.P., Jiang R.Y., Zhu J.Y., Sun K.N., Huang Y., Zhou H.H., Zheng Y.B., Wang X.J. (2024). PI3K/AKT/MTOR Signaling Pathway: An Important Driver and Therapeutic Target in Triple-Negative Breast Cancer. Breast Cancer.

[51] Agustiningsih A., Rasyak M.R., Turyadi, Jayanti S., Sukowati C. (2019). The Oncogenic Role of Hepatitis B Virus X Gene in Hepatocarcinogenesis: Recent Updates. Open Explor..

[52] Khorasani A.B.S., Delshad M., Sanaei M.J., Pourbagheri-Sigaroodi A., Pirsalehi A., Bashash D. (2025). Evaluating Oncogenic Drivers and Therapeutic Potential of the PI3K/AKT/MTOR Pathway in Hepatocellular Carcinoma: An Overview of Clinical Trials. Biocell.

[53] Kitab B., Kohara M., Tsukiyama-Kohara K., Rodrigo L., Martins I.J., Guo X., Qi X. (2021). Host-Targeting Antivirals for Treatment of Hepatitis C.

[54] Popa G.L., Popa M.I. (2022). Oxidative Stress in Chronic Hepatitis B—An Update. Microorganisms.

[55] Kostallari E., Schwabe R.F., Guillot A. (2025). Inflammation and Immunity in Liver Homeostasis and Disease: A Nexus of Hepatocytes, Nonparenchymal Cells and Immune Cells. Cell. Mol. Immunol..

[56] Wang X., Wei Z., Jiang Y., Meng Z., Lu M. (2021). MTOR Signaling: The Interface Linking Cellular Metabolism and Hepatitis B Virus Replication. Virol. Sin..

[57] Roncato R., Angelini J., Pani A., Talotta R. (2022). Lipid Rafts as Viral Entry Routes and Immune Platforms: A Double-Edged Sword in SARS-CoV-2 Infection?. Biochim. Biophys. Acta Mol. Cell Biol. Lipids.

[58] Zhu Y., Song H., Xu F., Huang M., Tan G. (2025). HBc: The Multifunctional Architect of HBV Replication, Immune Evasion, and Therapeutic Innovation. Front. Immunol..

[59] Mei L., Sun H., Yan Y., Ji H., Su Q., Chang L., Wang L. (2024). MTOR Signaling: Roles in Hepatitis B Virus Infection and Hepatocellular Carcinoma. Int. J. Biol. Sci..

[60] Molinaro A., Becattini B., Mazzoli A., Bleve A., Radici L., Maxvall I., Sopasakis V.R., Molinaro A., Bäckhed F., Solinas G. (2019). Insulin-Driven PI3K-AKT Signaling in the Hepatocyte Is Mediated by Redundant PI3Kα and PI3Kβ Activities and Is Promoted by RAS. Cell Metab..

[61] Xiang K., Wang B. (2018). Role of the PI3K-AKT-mTOR pathway in hepatitis B virus infection and replication. Mol. Med. Rep..

[62] Iwamoto M., Saso W., Sugiyama R., Ishii K., Ohki M., Nagamori S., Suzuki R., Aizaki H., Ryo A., Yun J.H. (2019). Epidermal growth factor receptor is a host-entry cofactor triggering hepatitis B virus internalization. Proc. Natl. Acad. Sci. USA.

[63] Lin S., Zhang Y.-J. (2017). Interference of Apoptosis by Hepatitis B Virus. Viruses.

[64] Song Y., Yang X., Shen Y., Wang Y., Xia X., Zhang A. (2019). STAT3 signaling pathway plays importantly genetic and functional roles in HCV infection. Mol. Genet. Genom. Med..

[65] Ringelhan M., Schuehle S., van de Klundert M., Kotsiliti E., Plissonnier M.L., Faure-Dupuy S., Riedl T., Lange S., Wisskirchen K., Thiele F. (2024). HBV-Related HCC Development in Mice Is STAT3 Dependent and Indicates an Oncogenic Effect of HBx. JHEP Rep..

[66] Mavragani C.P., Crow M.K. (2025). Type I Interferons in Health and Disease: Molecular Aspects and Clinical Implications. Physiol. Rev..

[67] Yu S., Ge H., Li S., Qiu H.J. (2022). Modulation of Macrophage Polarization by Viruses: Turning Off/On Host Antiviral Responses. Front. Microbiol..

[68] Hashimoto S., Hashimoto A., Muromoto R., Kitai Y., Oritani K., Matsuda T. (2022). Central Roles of STAT3-Mediated Signals in Onset and Development of Cancers: Tumorigenesis and Immunosurveillance. Cells.

[69] Darweesh M., Mohammadi S., Rahmati M., Al-Hamadani M., Al-Harrasi A. (2025). Metabolic Reprogramming in Viral Infections: The Interplay of Glucose Metabolism and Immune Responses. Front. Immunol..

[70] Frost E.R., Ford E.A., Peters A.E., Reed N.L., McLaughlin E.A., Baker M.A., Lovell-Badge R., Sutherland J.M. (2020). Signal Transducer and Activator of Transcription (STAT) 1 and STAT3 Are Expressed in the Human Ovary and Have Janus Kinase 1-Independent Functions in the COV434 Human Granulosa Cell Line. Reprod. Fertil. Dev..

[71] Yuan J., Zhang R., Geng Z. (2025). Cancer-Associated Fibroblasts in Lymph Node Metastasis: Insights and Therapeutic Strategies. Chin. Med. J..

[72] Srivastava N., Saxena A.K. (2025). Novel Small Molecule Inhibitors of Cyclin-Dependent Kinases as Anticancer Agents. Curr. Med. Chem..

[73] Szilveszter R.M., Muntean M., Florea A. (2024). Molecular Mechanisms in Tumorigenesis of Hepatocellular Carcinoma and in Target Treatments—An Overview. Biomolecules.

[74] Cornall A., Mak J., Greenway A., Tachedjian G. (2013). HIV-1 Infection of T Cells and Macrophages Are Differentially Modulated by Virion-Associated Hck: A Nef-Dependent Phenomenon. Viruses.

[75] Gillman R., Wankell M., Sun E.J., Ben David M., Karamatic R., Palamuthusingam P., Field M.A., Schmitz U., Hebbard L. (2025). Transcriptomic Characterization of North Queensland Hepatocellular Carcinoma. Oncology.

[76] Huang L.Y., Chiu C.J., Hsing C.H., Hsu Y.H. (2022). Interferon Family Cytokines in Obesity and Insulin Sensitivity. Cells.

[77] Zhang Y., Song J., Li T., Peng J., Zhou H., Zong Z., Zhang Y., Song J., Zhang Y., Li T. (2023). Emerging Role of Neutrophil Extracellular Traps in Gastrointestinal Tumors: A Narrative Review. Int. J. Mol. Sci..

[78] Qu B., Brown R.J. (2021). Strategies to Inhibit Hepatitis B Virus at the Transcript Level. Viruses.

[79] Gǎlbǎu C.Ş., Irimie M., Neculau A.E., Dima L., Pogačnik da Silva L., Vârciu M., Badea M. (2024). The Potential of Plant Extracts Used in Cosmetic Product Applications—Antioxidants Delivery and Mechanism of Actions. Antioxidants.

[80] Pan Q., Xie Y., Zhang Y., Guo X., Wang J., Liu M., Zhang X.L. (2024). EGFR Core Fucosylation, Induced by Hepatitis C Virus, Promotes TRIM40-Mediated-RIG-I Ubiquitination and Suppresses Interferon-I Antiviral Defenses. Nat. Commun..

[81] Fujii Y.R. (2020). The Quantum MicroRNA Immunity in Human Virus-Associated Diseases: Virtual Reality of HBV, HCV and HIV-1 Infection, and Hepatocellular Carcinogenesis with AI Machine Learning. Res. Artic. Arch. Clin. Biomed. Res..

[82] Nie K., Gao Y., Chen S., Wang Z., Wang H., Tang Y., Su H., Lu F., Dong H., Fang K. (2023). Diosgenin Attenuates Non-Alcoholic Fatty Liver Disease in Type 2 Diabetes through Regulating SIRT6-Related Fatty Acid Uptake. Phytomedicine.

[83] Liu Y., Wang J., Chen J., Wu S., Zeng X., Xiong Q., Guo Y., Sun J., Song F., Xu J. (2022). Upregulation of MiR-520c-3p via Hepatitis B Virus Drives Hepatocellular Migration and Invasion by the PTEN/AKT/NF-ΚB Axis. Mol. Ther. Nucleic Acids.

[84] Scalbert A., Manach C., Morand C., Rémésy C., Jiménez L. (2005). Dietary Polyphenols and the Prevention of Diseases. Crit. Rev. Food Sci. Nutr..

[85] Tsai Y.F., Lai J.I., Liu C.Y., Hsi C.N., Hsu C.Y., Huang C.C., Feng C.J., Lin Y.S., Chao T.C., Chiu J.H. (2025). Correlation Between PIK3R1 Expression and Cell Growth in Human Breast Cancer Cell Line BT-474 and Clinical Outcomes. World J. Oncol..

[86] Kim H., Park C.K., Lee S.J., Rha S.Y., Park K.H., Lim H.Y. (2013). PIK3CA Mutations in Hepatocellular Carcinoma in Korea. Yonsei Med. J..

[87] Jiang Y., Han Q., Zhao H., Zhang J. (2021). The Mechanisms of HBV-Induced Hepatocellular Carcinoma. J. Hepatocell. Carcinoma.

[88] Zeng Z., Cao X., Guo H., Lai Y., Yang W., He Y., Sun J., Sun Z., Li D., Tan Y. (2026). SYK Activation Enhances Dendritic Cell Functions in Spontaneous Rheumatoid Arthritis. Inflammation.

[89] Daina A., Michielin O., Zoete V. (2017). SwissADME: A free web tool to evaluate pharmacokinetics, drug-likeness and medicinal chemistry friendliness of small molecules. Sci. Rep..

[90] Ghani S., Khan N., Sable H., Yao F., Shafiq M. (2025). Computational techniques for enhancing PK/PD modeling and simulation and ADMET prediction. Computational Methods in Medicinal Chemistry, Pharmacology, and Toxicology.

[91] Xiao Q., Liu Y., Shu X., Li Y., Zhang X., Wang C., He S., Li J., Li T., Liu T. (2025). Molecular mechanisms of viral oncogenesis in haematological malignancies: Perspectives from metabolic reprogramming, epigenetic regulation and immune microenvironment remodeling. Exp. Hematol. Oncol..

[92] Yan Y., Qiu Y., Davgadorj C., Zheng C. (2023). Corrigendum: Novel molecular therapeutics targeting signaling pathway to control hepatitis B viral infection. Front. Cell. Infect. Microbiol..

[93] PubChem. https://pubchem.ncbi.nlm.nih.gov/.

[94] SwissTargetPrediction. http://www.swisstargetprediction.ch/.

[95] GeneCards—Human Genes. Gene Database. Gene Search. https://www.genecards.org/.

[96] Venny 2.1.0. https://bioinfogp.cnb.csic.es/tools/venny/.

[97] Szklarczyk D., Kirsch R., Koutrouli M., Nastou K., Mehryary F., Hachilif R., Gable A.L., Fang T., Doncheva N.T., Pyysalo S. (2023). The STRING Database in 2023: Protein–Protein Association Networks and Functional Enrichment Analyses for Any Sequenced Genome of Interest. Nucleic Acids Res..

[98] Download Cytoscape. https://cytoscape.org/download.html.

[99] Ge S.X., Jung D., Jung D., Yao R. (2020). ShinyGO: A Graphical Gene-Set Enrichment Tool for Animals and Plants. Bioinformatics.

[100] Barretto A.J.B., Orda M.A., Tsai P.W., Tayo L.L. (2024). Analysis of Modular Hub Genes and Therapeutic Targets across Stages of Non-Small Cell Lung Cancer Transcriptome. Genes.

[101] Virology V.M.-C. (2025). Functional Insights Through Gene Ontology, Disease Ontology, and KEGG Pathway Enrichment. Computational Virology.

[102] Ni Y., Seffernick A.E., Onar-Thomas A., Pounds S.B. (2024). Computing Power and Sample Size for the False Discovery Rate in Multiple Applications. Genes.

[103] Enrichment Analysis. https://bamboo.genobank.org/enrichment.html.

[104] Kannan D.C., Radhakrishnan M.S., Sambathkumar D.R., Dhanaraja M.D., Muvendhiran M.S., Dharnisha M.N.J. (2024). A Review on Step into the Future: Python Prescription (PyRx) Transforms Virtual Drug Discovery with AI-Driven Tools. Afr. J. Biomed. Res..

[105] Fitrianingsih S.P., Kurniati N.F., Fakih T.M., Adnyana I.K. (2025). Integrating Network Pharmacology, Molecular Docking, and Molecular Dynamics to Explore the Antidiabetic Mechanism of *Physalis angulata* L.. Pharmacia.

[106] Shana E. (2023). Protein-Ligand Interactions: Its Biological Process and Molecular Choreography from Drug Development to Cell Signaling. Enzym. Eng..

[107] BIOVIA Discovery Studio. Dassault Systèmes. https://www.3ds.com/products/biovia/discovery-studio.

