# Supplementary material for: Repurposing Product Nkabinde for Hepatitis B Virus Therapy: A Network Pharmacology and Molecular Docking Investigation
Source: Pharmaceuticals (Basel). 2026 Apr 16;19(4):627. doi: 10.3390/ph19040627 (PMC13118322; doi:10.3390/ph19040627)
Supplement: Supplementary file 1 [file pharmaceuticals-19-00627-s001.zip › Figure S1-S10 2D diagram of Protein-ligand interactions of the 10 hub genes.pdf]

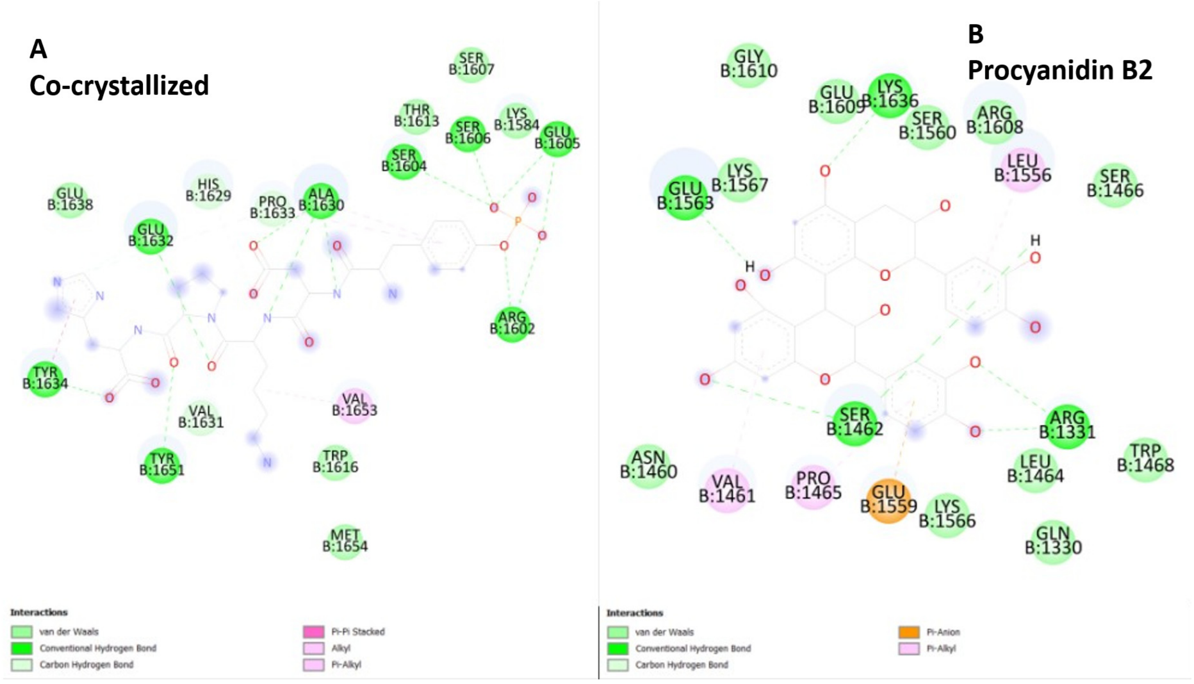

Figure S1. 2D diagram of the STAT1-Ligand complexes

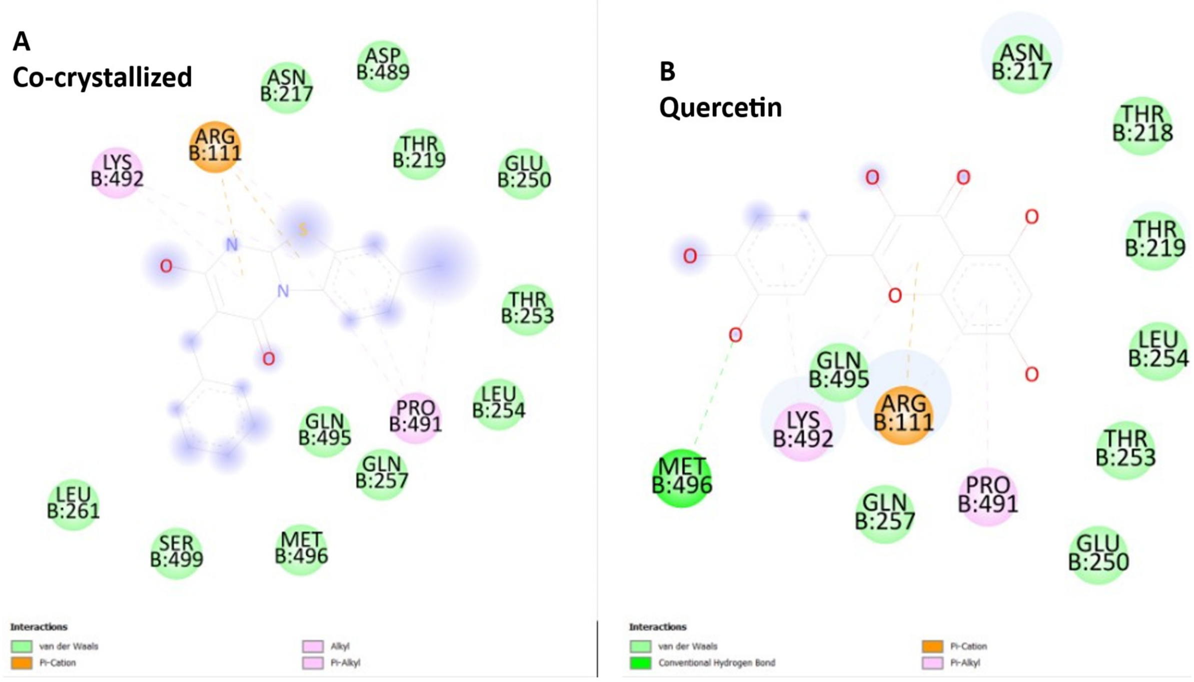

Figure S2. 2D diagram of the PTPN11-Ligand complexes

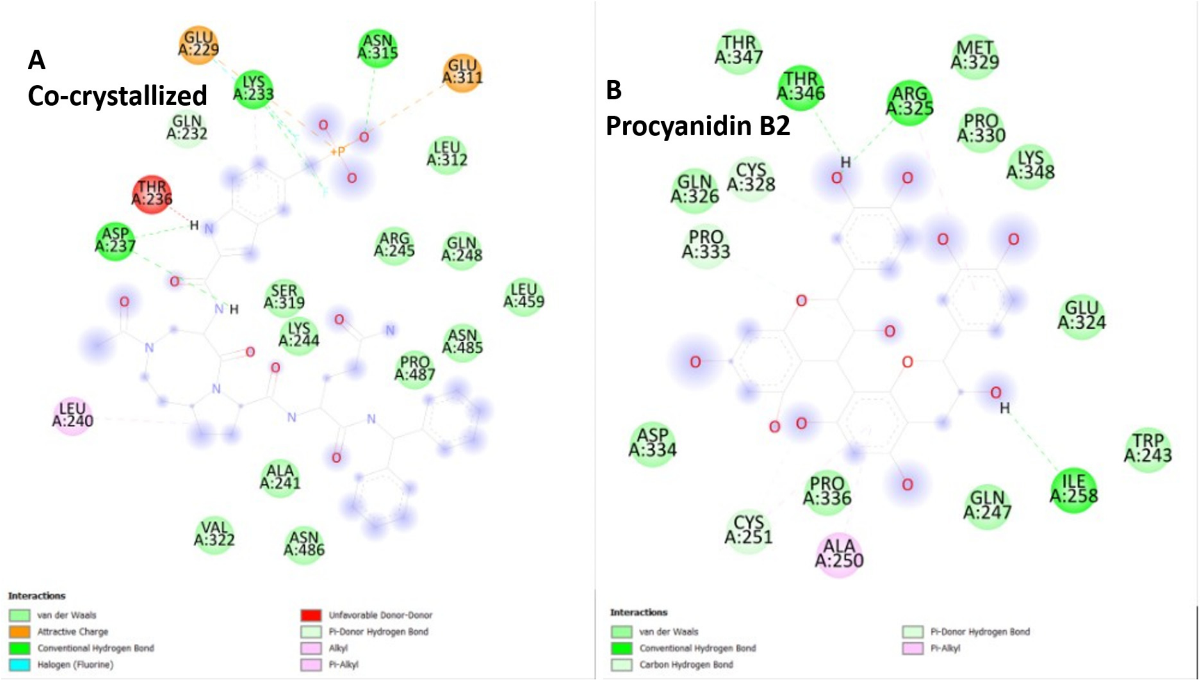

Figure S3. 2D diagram of the STAT3-Ligand complexes

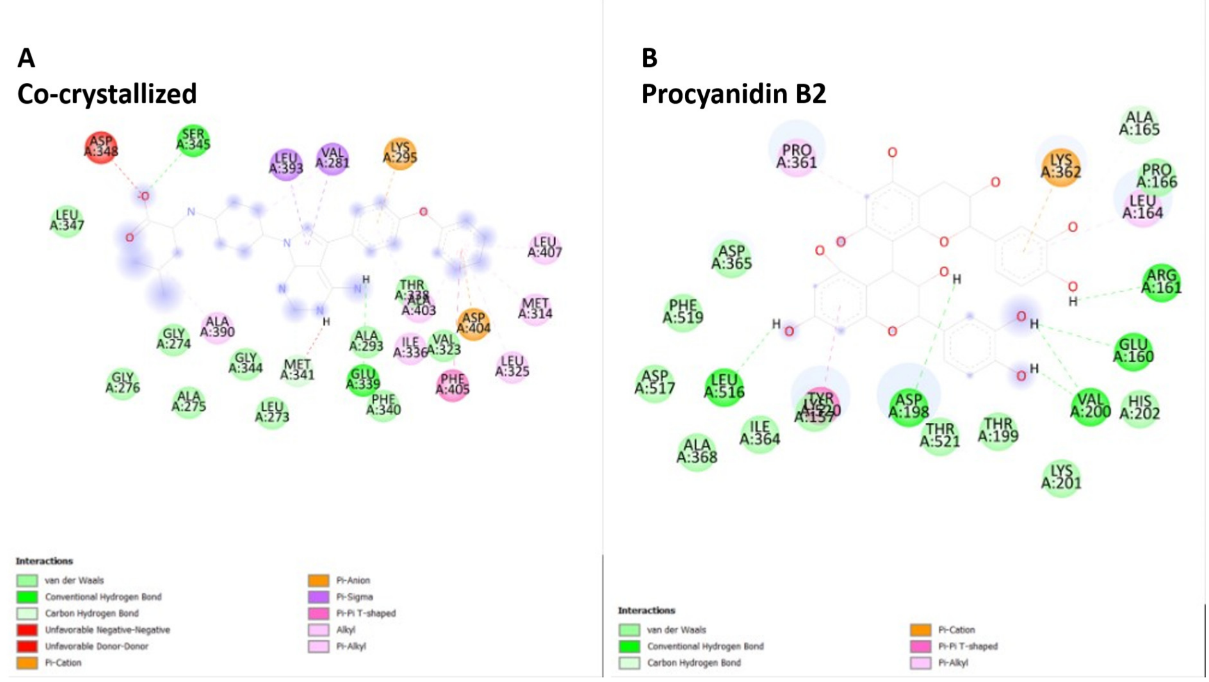

Figure S4. 2D diagram of the HCK-Ligand complexes

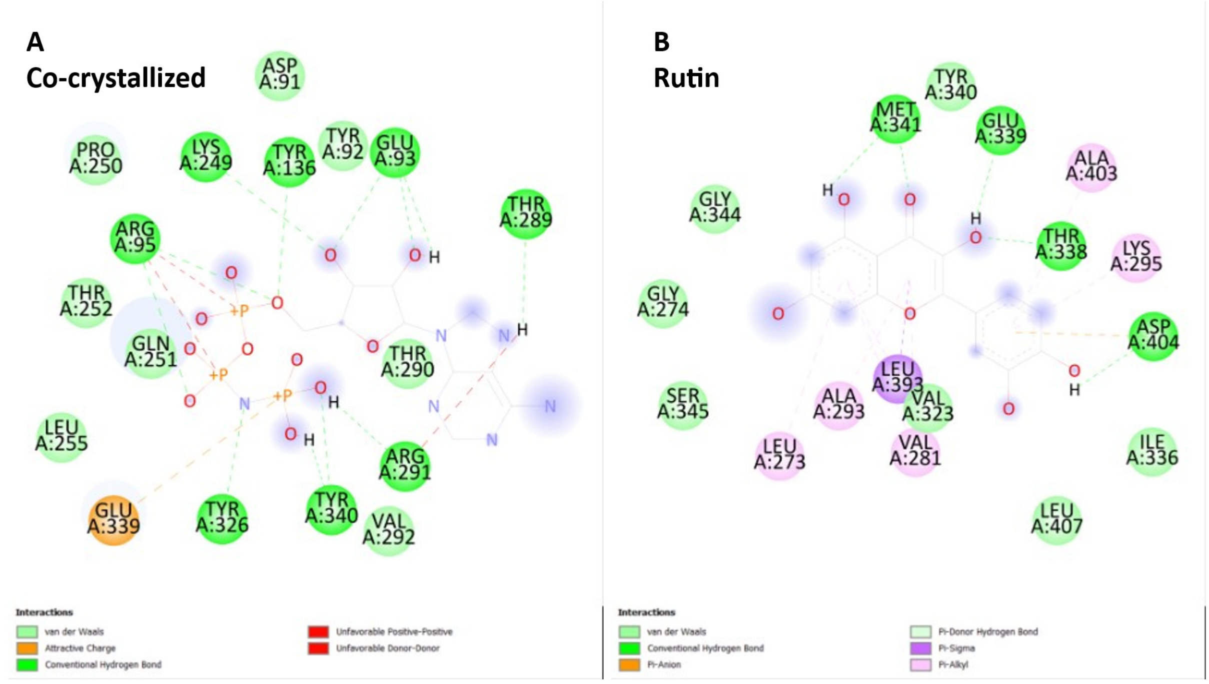

Figure S5. 2D diagram of the SRC-Ligand complexes

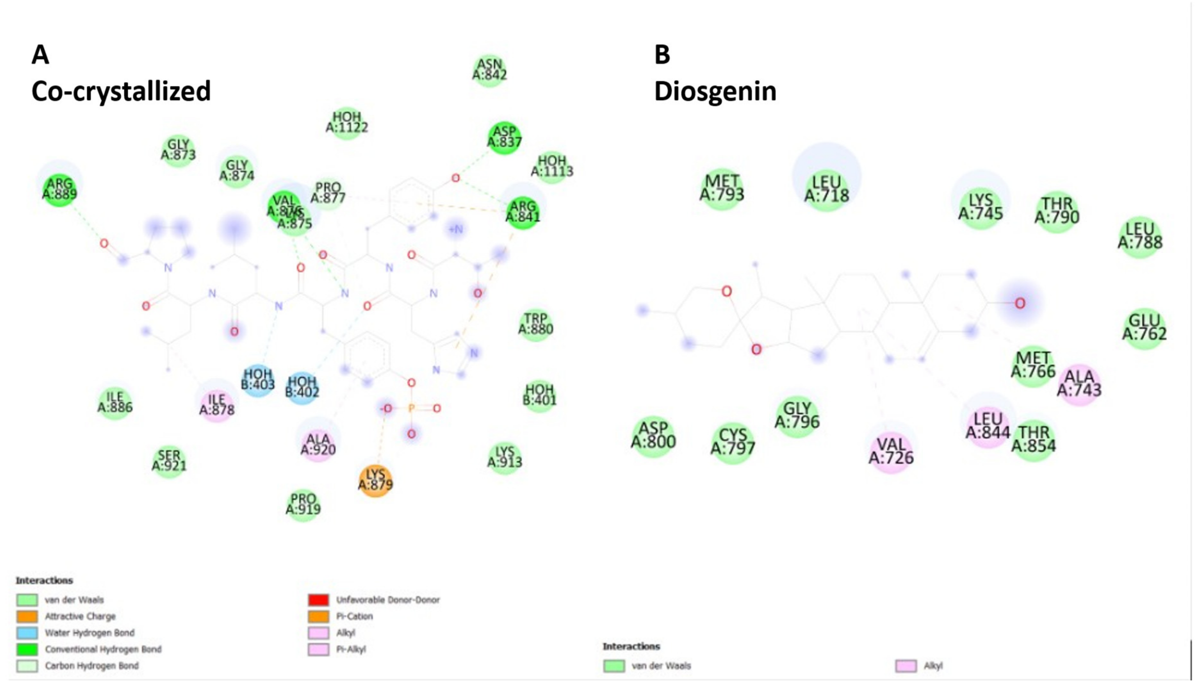

Figure S6. 2D diagram of the EGFR-Ligand complexes

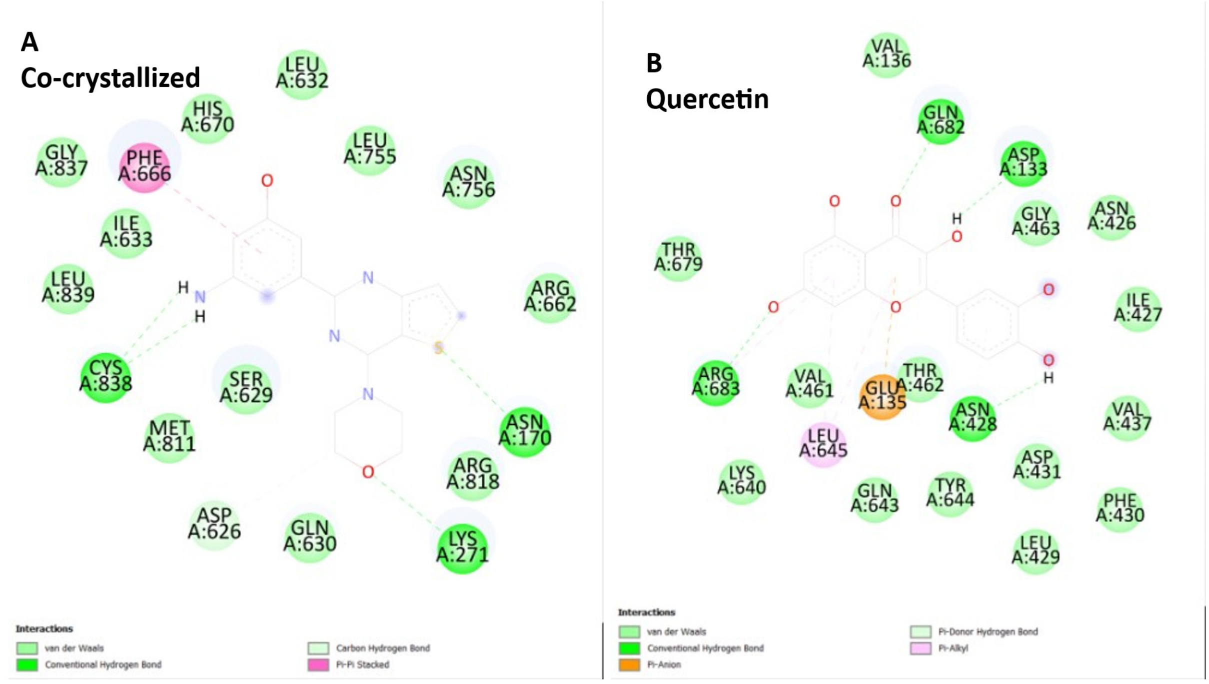

Figure S7. 2D diagram of the PIK3R1-Ligand complexes

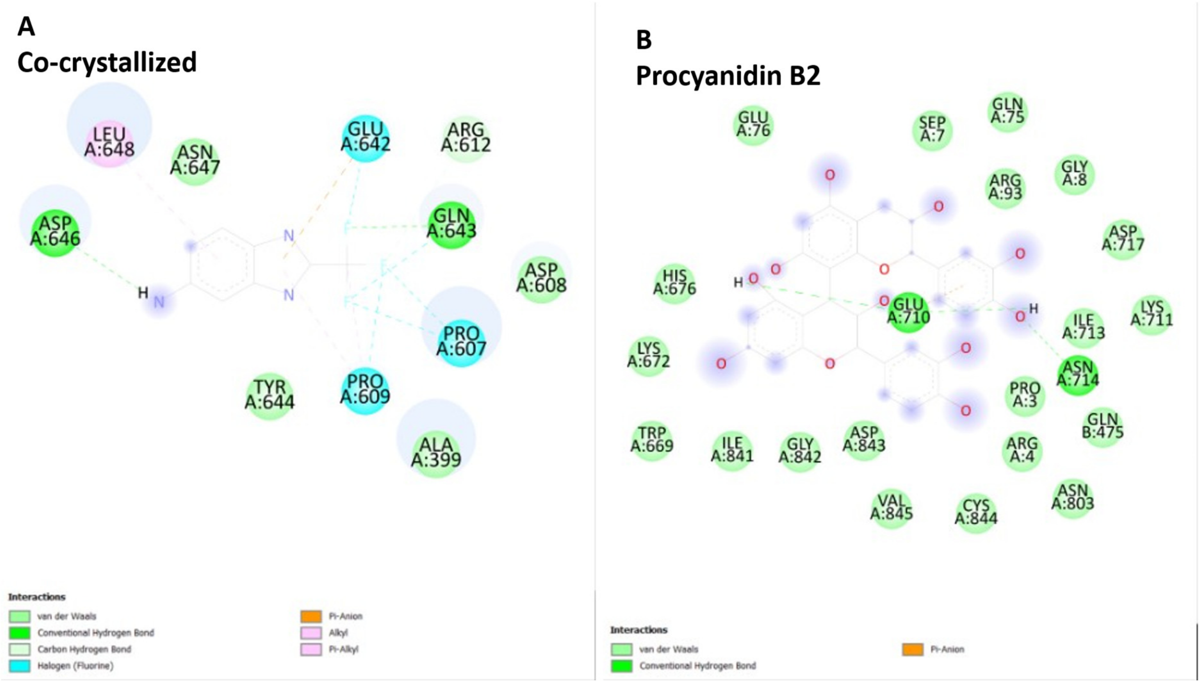

Figure S8. 2D diagram of the PIK3CA-Ligand complexes

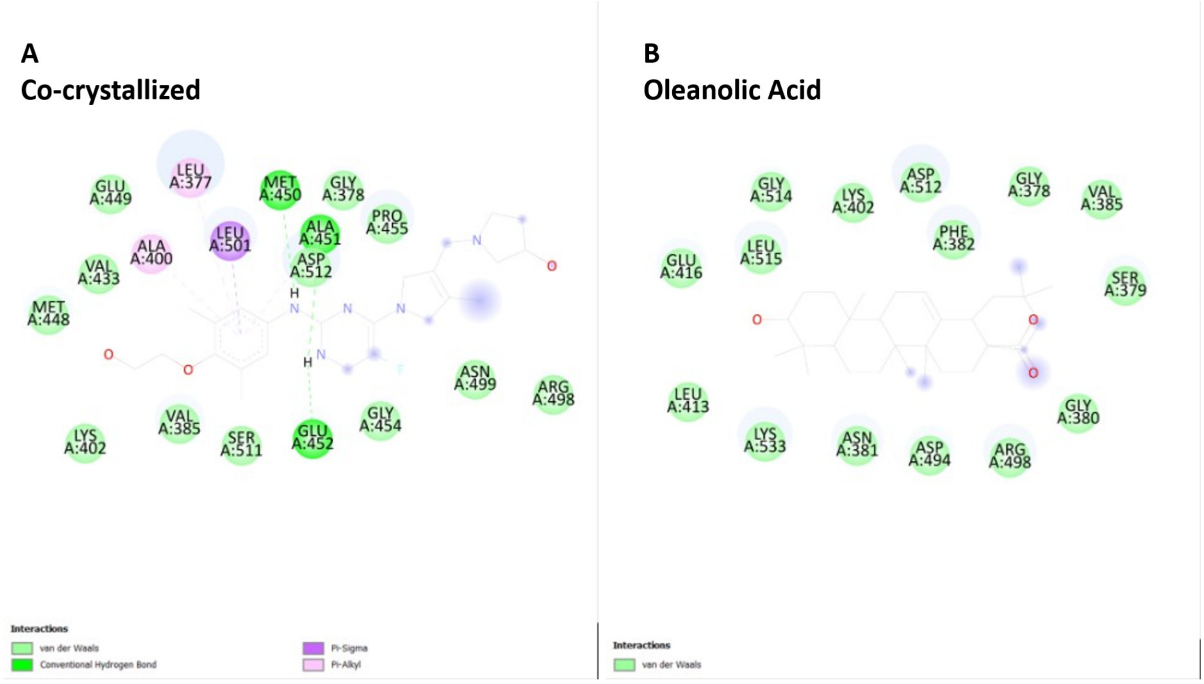

Figure S9. 2D diagram of the SYK-Ligand complexes

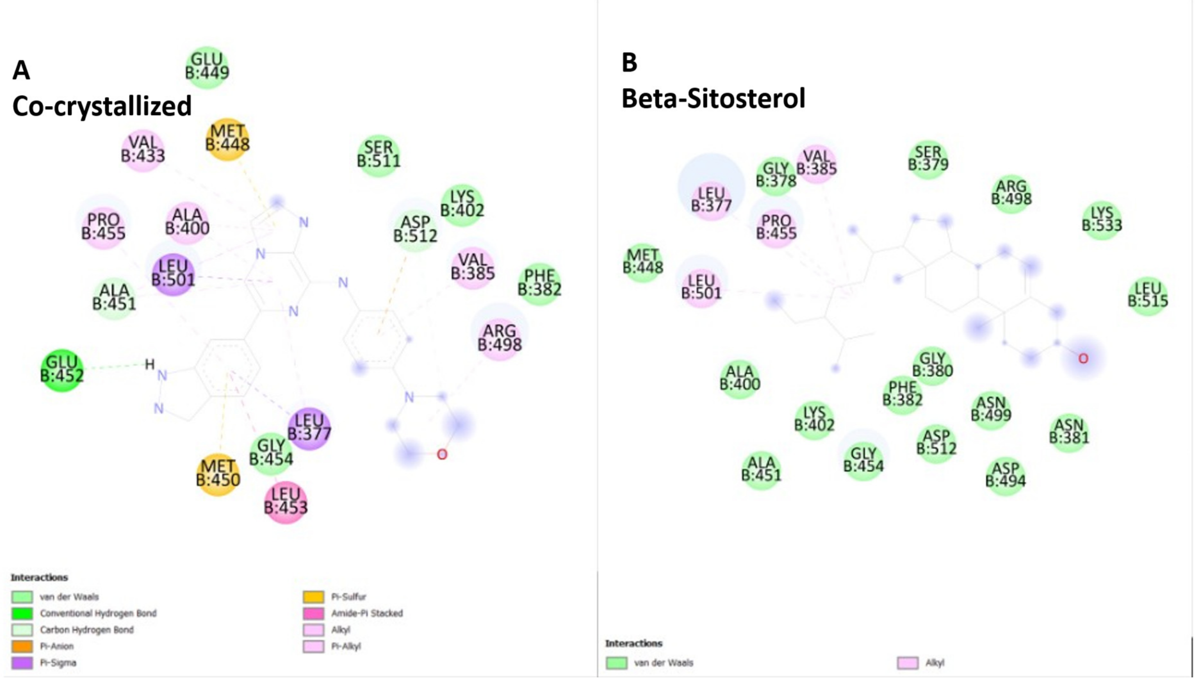

Figure S10. 2D diagram of the PIK3CB-Ligand complexes
