# Supplementary material for: Repurposing Product Nkabinde for Hepatitis B Virus Therapy: A Network Pharmacology and Molecular Docking Investigation
Source: Pharmaceuticals (Basel). 2026 Apr 16;19(4):627. doi: 10.3390/ph19040627 (PMC13118322; doi:10.3390/ph19040627)
Supplement: Supplementary file 1 [file pharmaceuticals-19-00627-s001.zip › Supplementary Figures S12-S21_Superimposed structures.pdf]

Supplementary Figures 12-21: The original cocrystallized ligand is in green, while the redocked cocrystallized ligand is in magenta.

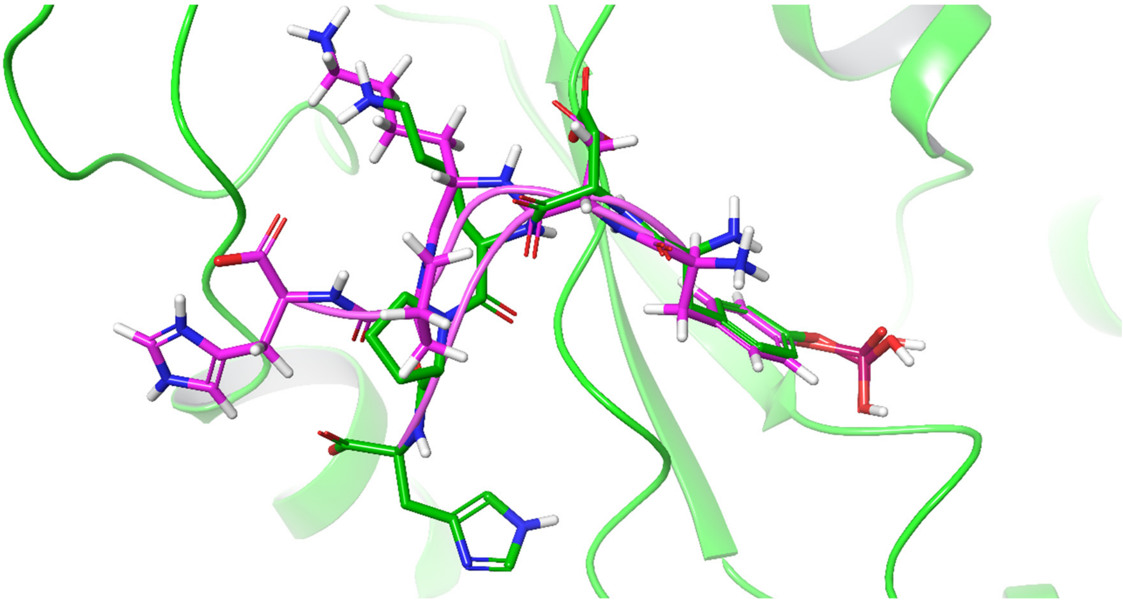

Figure S12. STAT1

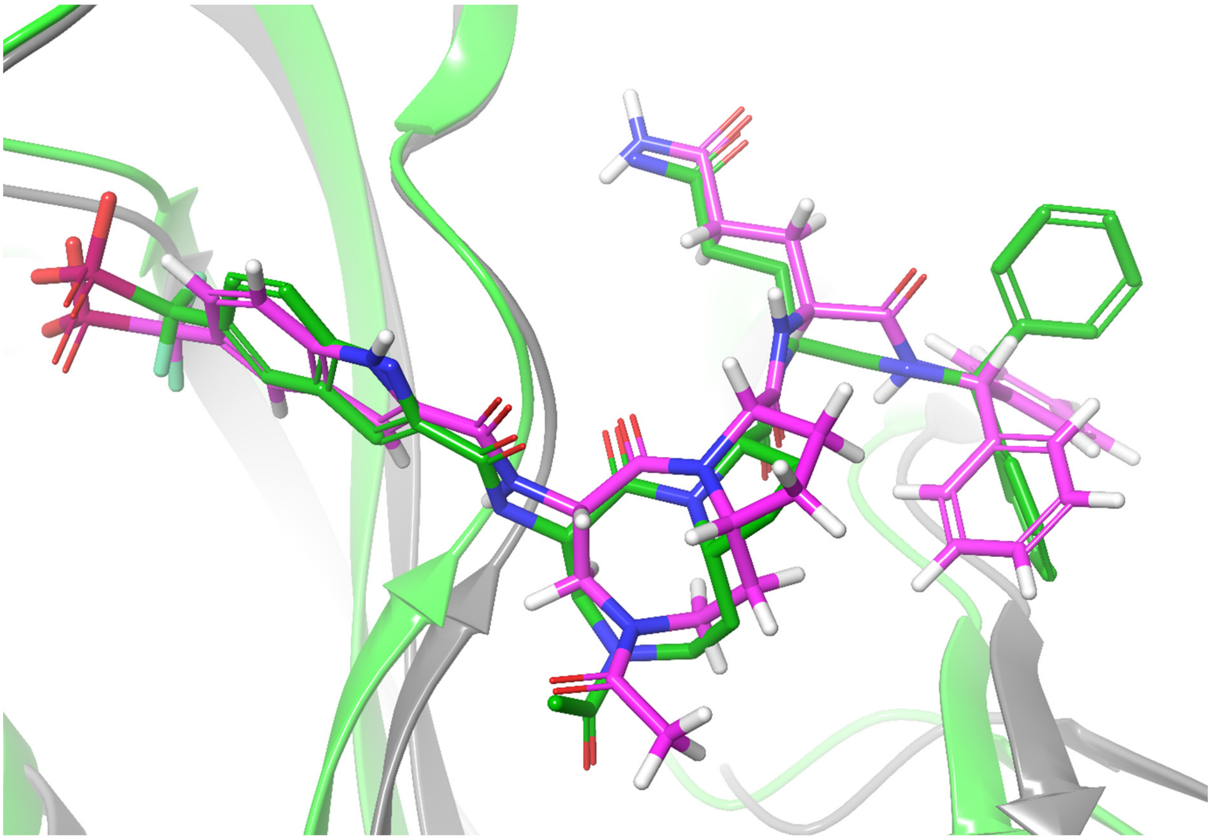

Figure S13. STAT3

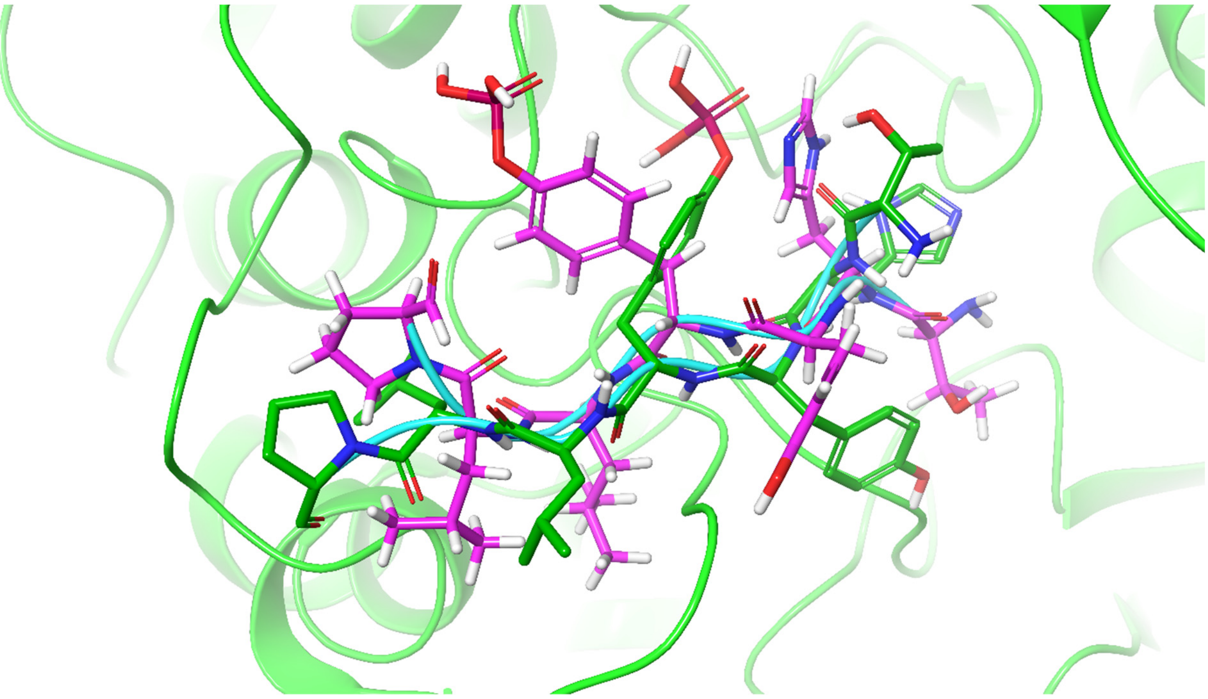

Figure S14.EGFR

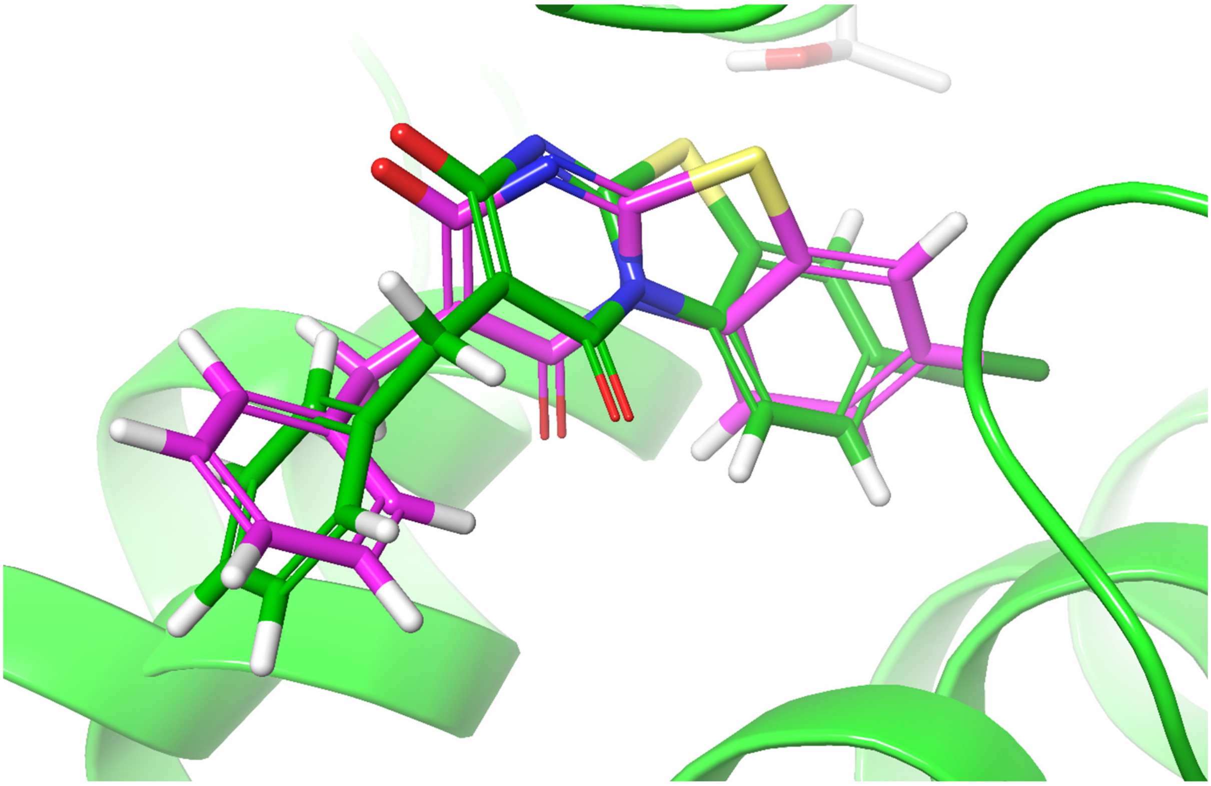

Figure S15. PTPN11

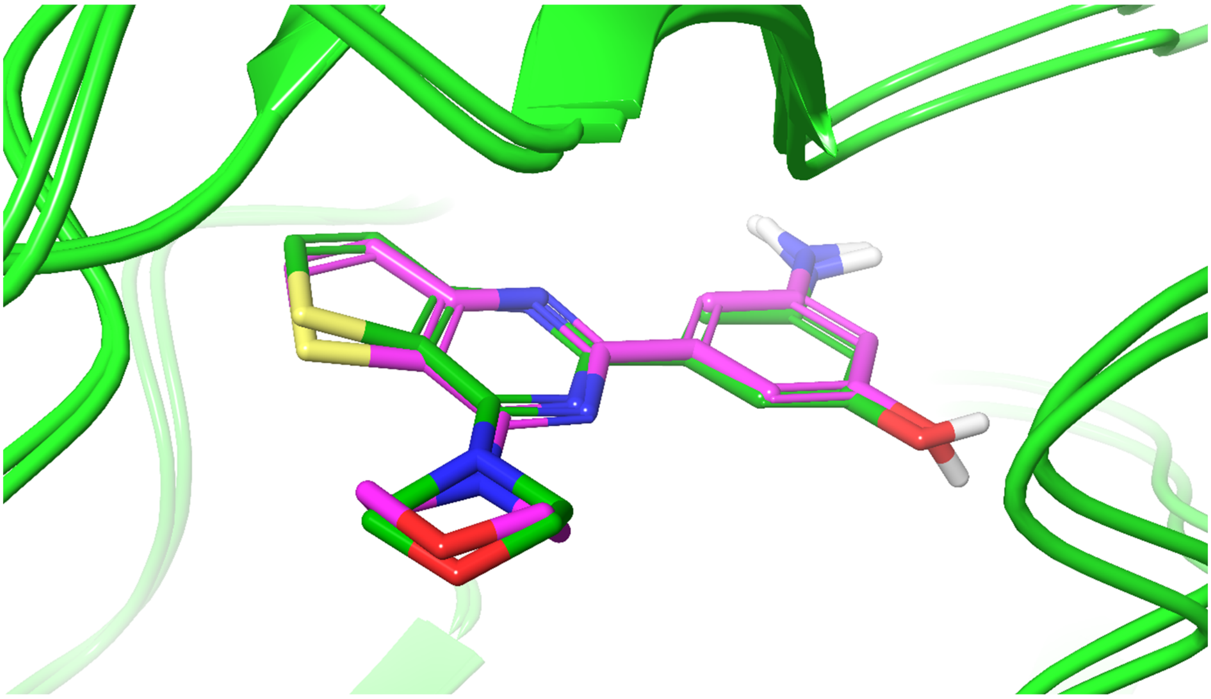

Figure S16. PIK3R1

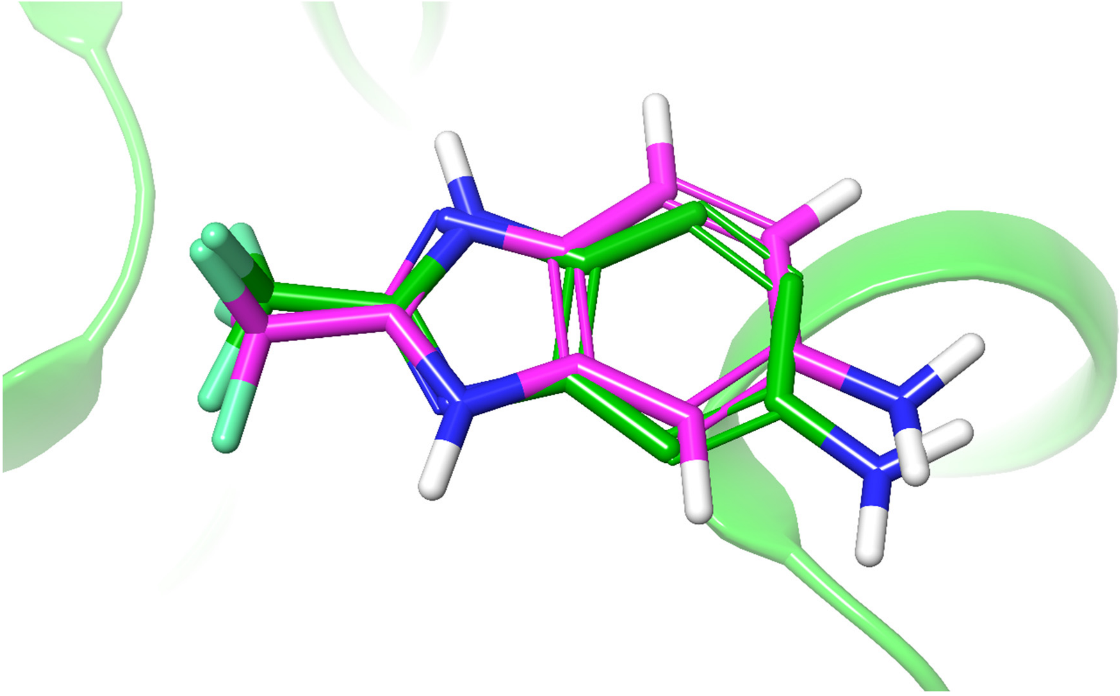

Figure S17. PIK3CA

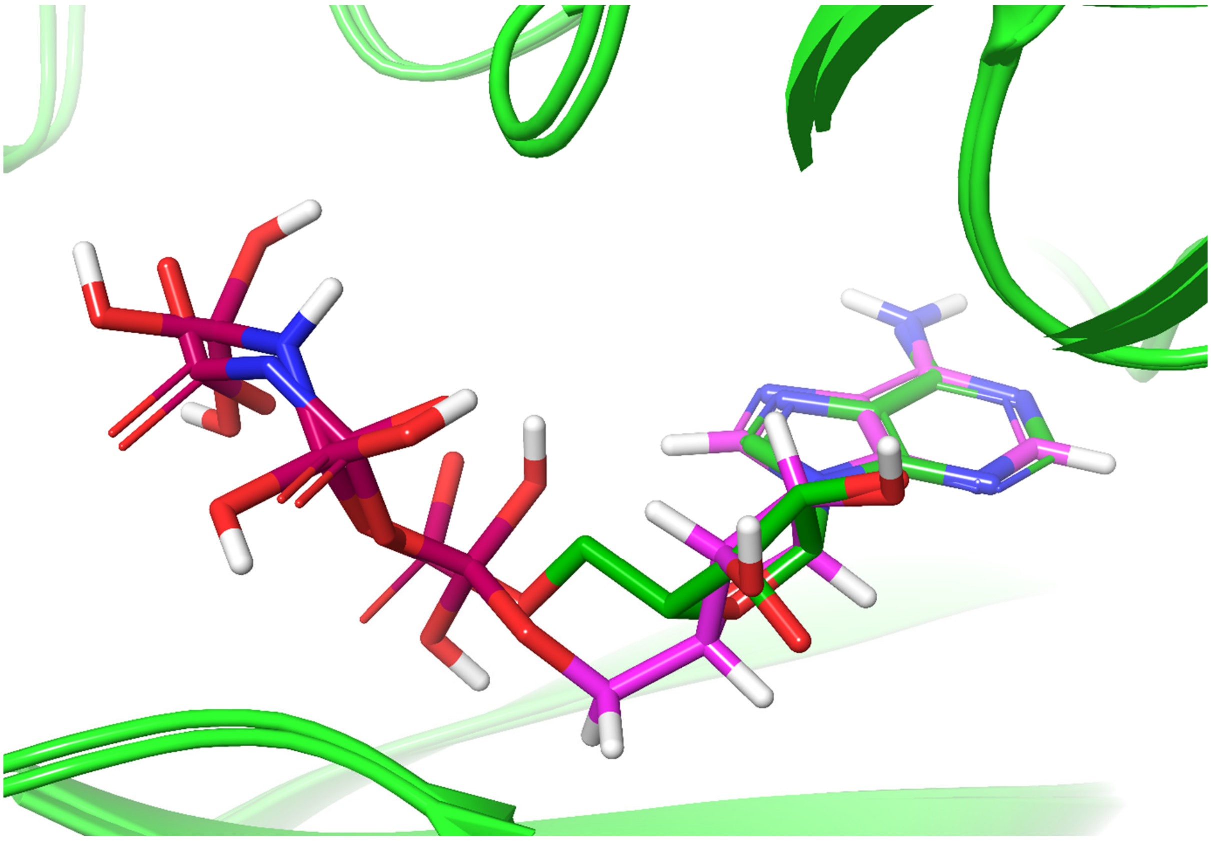

Figure S18. SRC

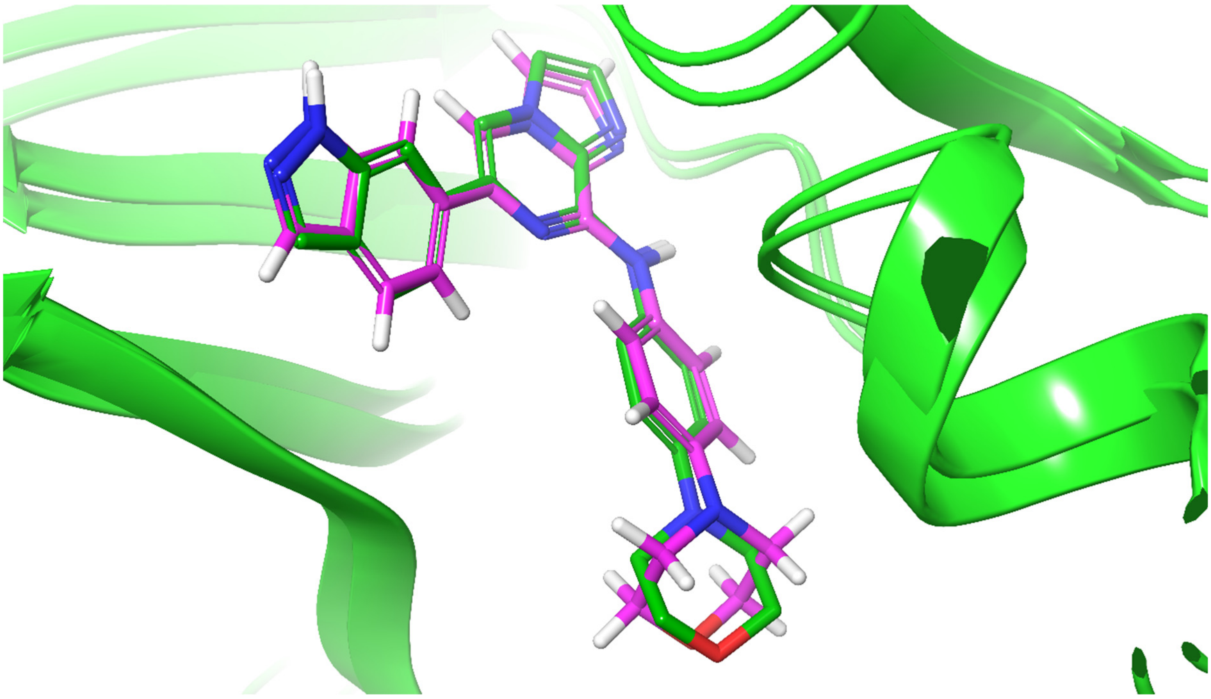

Figure S19. PIK3CB

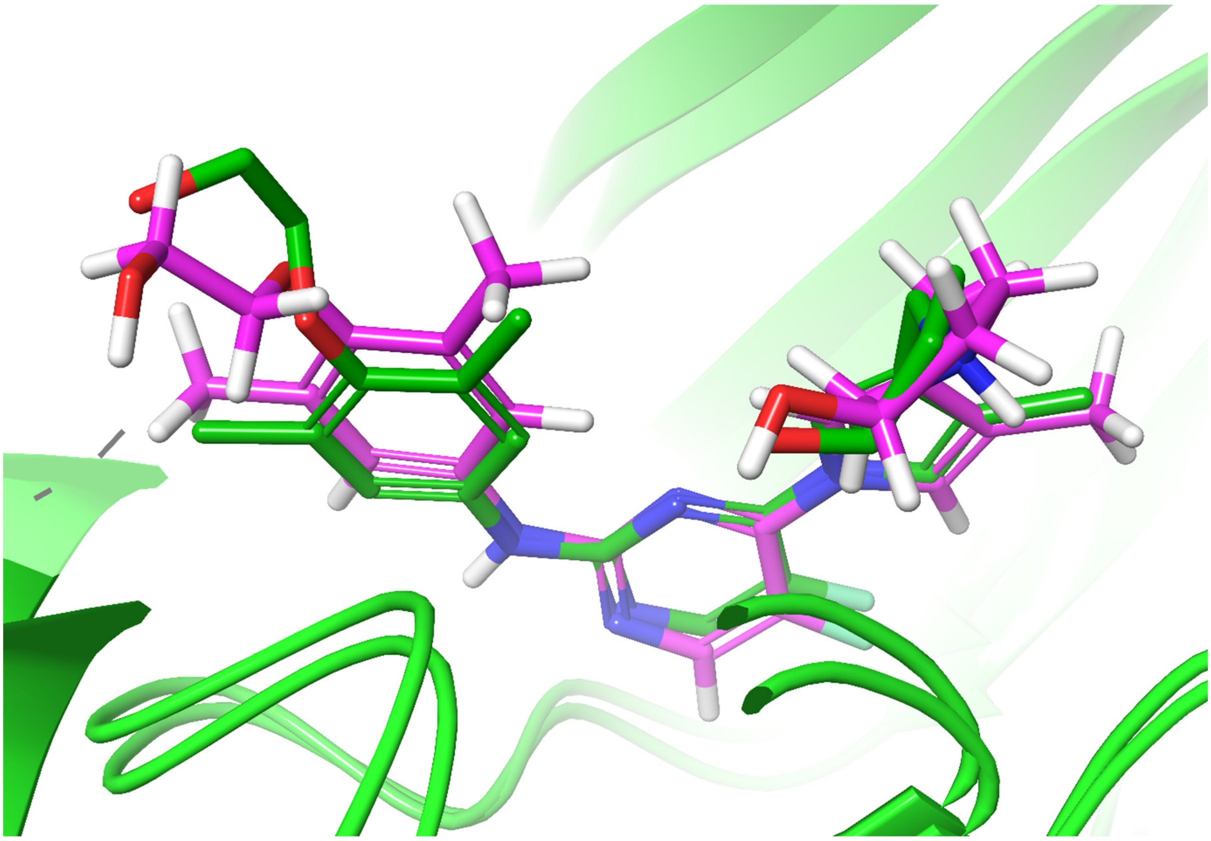

Figure S20. SYK

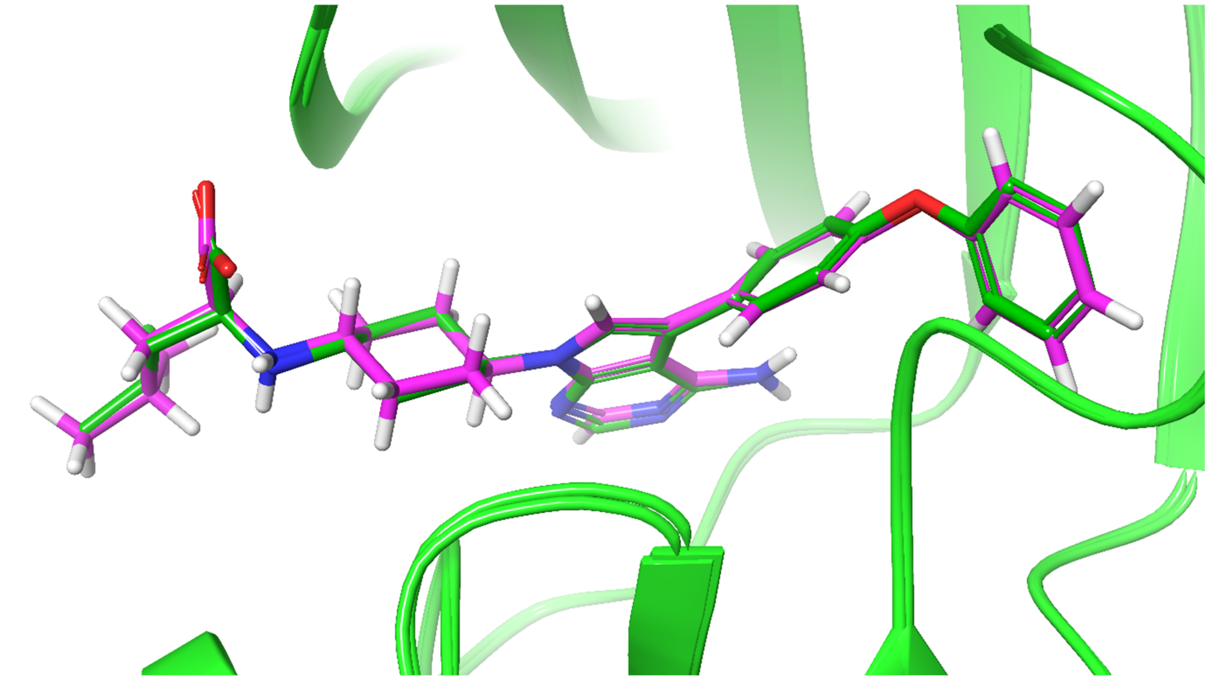

Figure 21. HCK
