# Supplementary material for: Repurposing Product Nkabinde for Hepatitis B Virus Therapy: A Network Pharmacology and Molecular Docking Investigation
Source: Pharmaceuticals (Basel). 2026 Apr 16;19(4):627. doi: 10.3390/ph19040627 (PMC13118322; doi:10.3390/ph19040627)
Supplement: Supplementary file 1 [file pharmaceuticals-19-00627-s001.zip › Supplementary Table S3_All the docking results.pdf]

PN Phytochemical	STAT1	STAT3	SRC	EGFR	PTPN11	HCK	PIK3CA	PIK3CB	PIK3R1	SYK
quercetin-3-O-β-D-(6′-galloyl)-glucopyranoside	−8.6	−8.6	−10.6	−8.3	−8.1	−10.7	−8.3	−8.7	−10.2	−8.2
Diosgenin	−8.9	−8.6	−10.0	−9.4	−9.2	−10.6	−10.7	−9.9	−10.5	−8.7
Prostratin	−7.9	−7.6	−8.4	−7.8	−7.5	−8.1	−8.3	−7.8	−8.0	−7.6
6-(8″-Umbelliferyl)-apigenin	−8.2	−8.3	−10.6	−8.7	−8.1	−10.7	−8.9	−8.2	−10.2	−8.1
Pimelea factor P2	−8.7	−8.4	−10.3	−8.3	−8.2	−10.0	−9.5	−8.7	−11.0	−8.7
Wikstroelide A	−8.1	−7.9	−9.4	−8.0	−8.0	−9.6	−9.0	−7.9	−9.8	−7.8
Gnidicin	−8.5	−8.5	−10.2	−8.9	−8.9	−10.0	−9.1	−9.1	−10.4	−9.4
Gnidilatidin	−8.4	−8.3	−9.9	−8.5	−8.6	−9.8	−9.2	−8.1	−9.9	−8.3
Gnidimacrin	−9.0	−8.5	−10.2	−8.6	−8.6	−9.8	−8.7	−9.2	−10.1	−8.5
(−)-Epicatechin	−8.0	−7.8	−8.9	−7.9	−7.7	−9.0	−8.1	−7.9	−8.8	−7.8
Oleanolic acid	−8.8	−8.2	−10.0	−8.0	−8.9	−10.3	−10.3	−8.9	−10.3	−14.0
Procyanidin B2	−9.0	−8.7	−10.2	−8.3	−8.1	−10.5	−9.5	−8.5	−10.1	−8.4
Epigallocatechin gallate	−8.7	−8.7	−9.6	−8.1	−8.0	−9.9	−9.7	−8.2	−9.8	−8.7
Quercetin	−8.4	−8.2	−9.8	−8.0	−9.2	−9.6	−9.1	−8.1	−10.6	−8.0
Catechin	−8.1	−7.9	−9.0	−7.8	−7.7	−9.1	−8.4	−7.9	−8.9	−7.8
Emodin	−8.2	−8.0	−9.4	−7.9	−8.0	−9.3	−9.1	−7.8	−9.5	−7.9
Daucosterol	−7.9	−7.6	−8.8	−7.5	−7.6	−9.0	−9.5	−8.5	−9.2	−7.6
β-Sitosterol	−8.0	−7.7	−9.1	−7.6	−7.8	−9.2	−8.4	−14.2	−9.0	−7.7
Rutin	−8.6	−8.7	−10.5	−8.7	−8.6	−10.6	−9.1	−8.6	−10.6	−8.7
Chrysophanol	−7.8	−7.6	−8.9	−7.7	−7.6	−9.0	−8.8	−7.9	−8.9	−7.6
Physcion	−7.9	−7.7	−9.0	−7.8	−7.7	−9.1	−8.0	−7.9	−9.0	−7.7
7,7′-dihydroxy-3,8′-biscoumarin	-8.1	-8.3	-8.2	-8.0	-9.1	-9.2	-8.1	-7.9	-9.2	-8.0
Aloin	−8.0	−7.9	−9.2	−7.9	−7.8	−9.3	−9.5	−7.9	−9.4	−8.7
Gallic acid	−6.9	−6.7	−7.8	−6.8	−6.9	−8.0	−6.3	−5.7	−7.9	−6.8
Quercetin-3-O-arabinoside	−8.3	−8.2	−9.9	−8.0	−8.1	−9.8	−9.2	−8.4	−9.9	−8.2
2,4′,6-trihydroxy-4-methoxybenzophenone-2-O-glucoside	−8.1	−7.9	−9.4	−7.8	−7.9	−9.3	−8.9	−7.9	−9.5	−7.8
2,3,4′,5,6-pentahydroxybenzophenone-4-C-glucoside	−8.0	−7.8	−9.2	−7.7	−7.8	−9.1	−8.7	−7.8	−9.3	−7.7
