# Supplementary material for: Repurposing Product Nkabinde for Hepatitis B Virus Therapy: A Network Pharmacology and Molecular Docking Investigation
Source: Pharmaceuticals (Basel). 2026 Apr 16;19(4):627. doi: 10.3390/ph19040627 (PMC13118322; doi:10.3390/ph19040627)
Supplement: Supplementary file 1 [file pharmaceuticals-19-00627-s001.zip › Supplementary_Table_S5_ADMET_PN.pdf]

Supplementary Table S5. In silico ADMET and drug-likeness profiling of Product Nkabinde phytochemicals (SwissADME)

Phytochemical	MW (g/mol)	TPSA	XlogP3	HBD	HBA	Rot Bonds	Lipinski Violation	GI Abs	BBB	P-gp	Bioavailability
Quercetin-3-O-β-D-(6′-galloyl)-glucopyranoside	616.48	277.27	0.98	10	16	7	3	Low	No	No	0.17
7,7′-Dihydroxy-3,8′-biscoumarin	322.27	100.88	2.81	2	6	1	0	High	No	No	0.55
Prostratin	390.47	104.06	0.7	3	6	3	0	High	No	Yes	0.55
6-(8″-Umbelliferyl)-apigenin	322.27	100.88	2.46	2	6	1	0	High	No	No	0.55
Pimelea factor P2	638.79	127.21	6.15	3	9	5	1	Low	No	Yes	0.55
Wikstroelide A	642.78	144.28	5.33	3	10	14	1	Low	No	Yes	0.55
Gnidicin	628.67	144.28	3.16	3	10	7	1	Low	No	Yes	0.55
Gnidilatidin	648.74	144.28	4.82	3	10	11	1	Low	No	Yes	0.55
Gnidimacrin	774.89	173.74	6.01	4	12	9	2	Low	No	Yes	0.17
(−)-Epicatechin	290.27	110.38	0.36	5	6	1	0	High	No	Yes	0.55
Diosgenin	414.62	38.69	5.67	1	3	0	1	High	Yes	No	0.55
Oleanolic acid	456.7	57.53	7.49	2	3	1	1	Low	No	No	0.85
Procyanidin B2	578.52	220.76	2.37	10	12	3	3	Low	No	No	0.17
Epigallocatechin gallate	458.37	197.37	1.17	8	11	4	2	Low	No	No	0.17
Quercetin	302.24	131.36	1.54	5	7	1	0	High	No	No	0.55
Catechin	290.27	110.38	0.36	5	6	1	0	High	No	Yes	0.55
Emodin	270.24	94.83	2.72	3	5	0	0	High	No	No	0.55
Daucosterol	576.85	99.38	7.74	4	6	9	1	Low	No	No	0.55
β-Sitosterol	414.71	20.23	9.34	1	1	6	1	Low	No	No	0.55
Rutin	610.52	269.43	-0.33	10	16	6	3	Low	No	Yes	0.17
Chrysophanol	254.24	74.6	3.53	2	4	0	0	High	Yes	No	0.55
Physcion	284.26	83.83	3.04	2	5	1	0	High	No	No	0.55
Gallic acid	170.12	97.99	0.7	4	5	1	0	High	No	No	0.56
Quercetin-3-O-arabinoside	434.35	190.28	0.43	7	11	3	2	Low	No	No	0.17
Aloin	418.39	167.91	-0.12	7	9	3	1	Low	No	No	0.55
2,4′,6-Trihydroxy-4-methoxybenzophenone-2-O-glucoside	430.36	141.34	3.92	4	8	2	0	Low	No	No	0.55
2,3,4′,5,6-Pentahydroxybenzophenone-4-C-glucoside	422.38	166.14	0.66	6	10	6	1	Low	No	No	0.55
