# Supplementary material for: Repurposing Product Nkabinde for Hepatitis B Virus Therapy: A Network Pharmacology and Molecular Docking Investigation
Source: Pharmaceuticals (Basel). 2026 Apr 16;19(4):627. doi: 10.3390/ph19040627 (PMC13118322; doi:10.3390/ph19040627)
Supplement: Supplementary file 1 [file pharmaceuticals-19-00627-s001.zip › Table_S1_Docking_Grid_Parameters (2).pdf]

Table S1. AutoDock Vina grid box parameters used for redocking and phytochemical docking

Target Protein	PDB ID	Grid Center (Å)	Grid Size (Å)	Exhaustiveness	Number of Poses
STAT1	1YVL	X = −9.4601<br>Y =−46.2330<br>Z = 193.8382	X = 99.8062<br>Y = 58.9802<br>Z = 115.5129	8	9
STAT3	6NUQ	X = −2.2294<br>Y = 19.1326<br>Z = 24.6217	X = 72.3191<br>Y = 115.0291<br>Z = 91.5312	8	9
PIK3CA	5SXA	X = 68.7928<br>Y = 58.3057<br>Z = 93.1120	X = 121.8861<br>Y = 108.4890<br>Z = 98.4729	8	9
PIK3CB	4PUZ	X = −30.1911<br>Y = −0.2475<br>Z = 58.7412	X = 45.8255<br>Y = 55.3426<br>Z = 60.3925	8	9
PIK3R1	5XGI	X = 17.8179<br>Y = 34.1451<br>Z = 30.0453	X = 88.4854<br>Y = 111.0938<br>Z = 101.1489	8	9
EGFR	4R3P	X = −57.2821<br>Y = −7.9282<br>Z = −24.8951	X = 50.3781<br>Y = 64.6770<br>Z = 56.2785	8	9
SRC	2SRC	X = 20.6723<br>Y = 33.8852<br>Z = 67.4994	X = 64.5127<br>Y = 68.7439<br>Z = 60.9835	8	9
HCK	5H0B	X = 5.7224<br>Y = −2.5091<br>Z = −15.9866	X = 59.6835<br>Y = 81.7633<br>Z = 73.4306	8	9
SYK	4XG4	X = 12.9037<br>Y = −11.6072<br>Z = 17.6213	X = 46.9783<br>Y = 59.2202<br>Z = 55.6463	8	9
PTPN11	6BN5	X = 64.0642<br>Y = 91.0075<br>Z = 17.1150	X = 60.5899<br>Y = 74.2658<br>Z = 60.3082	8	9
