# Supplementary material for: Repurposing Product Nkabinde for Hepatitis B Virus Therapy: A Network Pharmacology and Molecular Docking Investigation
Source: Pharmaceuticals (Basel). 2026 Apr 16;19(4):627. doi: 10.3390/ph19040627 (PMC13118322; doi:10.3390/ph19040627)
Supplement: Supplementary file 1 [file pharmaceuticals-19-00627-s001.zip › Table_S2_Redocking_Validation.pdf]

Table S2. RMSD redocking validation results of co-crystallized ligands

Target Protein	PDB ID	Docking Score (kcal/mol)	RMSD (Å)
STAT1	1YVL	-10.8	0.125
STAT3	6NUQ	-8.9	0.081
PIK3CA	5SXA	-7.3	0.099
PIK3CB	4PUZ	-7.9	0.083
PIK3R1	5XGI	-8.2	0.093
EGFR	4R3P	-8.4	0.087
SRC	2SRC	-7.8	0.116
SYK	4XG4	-8.3	0.092
HCK	5H0B	-7.9	0.094
PTPN11	6BN5	-7.5	0.101
