# Supplementary figures and images for: Repurposing Product Nkabinde for Hepatitis B Virus Therapy: A Network Pharmacology and Molecular Docking Investigation
Source: Pharmaceuticals (Basel). 2026 Apr 16;19(4):627. doi: 10.3390/ph19040627 (PMC13118322; doi:10.3390/ph19040627)

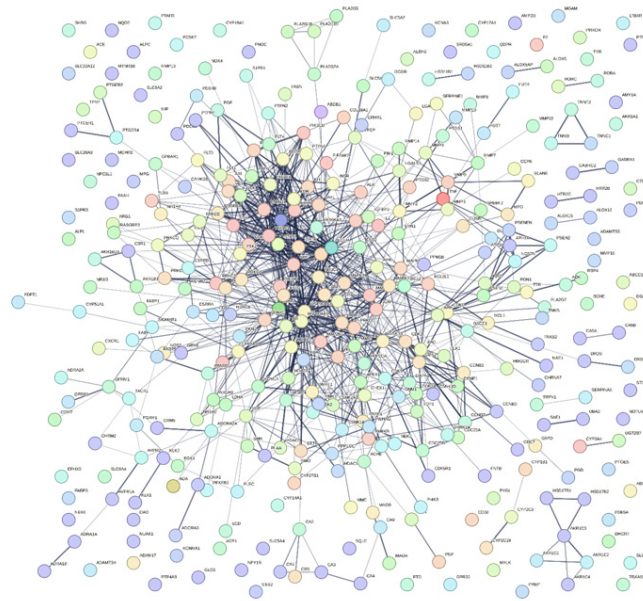

Figure S11. Interaction Network of the 359 PN-HBV common genes

Supplement: Supplementary file 1 [file pharmaceuticals-19-00627-s001.zip › Supplementary Figure S11.pdf]
